# GRP78 in human diseases: From molecular chaperone to therapeutic target

**DOI:** 10.7150/thno.136060

**Published:** 2026-05-29

**Authors:** Yang Li, Dan Mu, Jiajie Feng, Zhijia Li, Lan Zhang, Lei Wang

**Affiliations:** 1 Department of Hematology, Shengjing Hospital of China Medical University, Shenyang 110001, China.; 2 Sichuan Engineering Research Center for Biomimetic Synthesis of Natural Drugs, School of Life Science and Engineering, Southwest Jiaotong University, Chengdu 610031, China.; 3 Department of Hematology, The First Hospital of China Medical University, Shenyang 110001, China.

**Keywords:** GRP78, molecular chaperone, post-translational modifications, structure and function, human diseases, targeted therapy

## Abstract

Glucose-regulated Protein 78 (GRP78, also known as BiP/HSPA5) is a central member of the Hsp70 family. As a key molecular chaperone in the endoplasmic reticulum (ER), it plays an important role in cell survival and biological function by maintaining protein folding homeostasis and regulating endoplasmic reticulum stress (ERS) and the unfolded protein response (UPR). Its function is precisely regulated by various post-translational modifications (PTMs), including phosphorylation and acetylation. In addition, GRP78 can translocate to subcellular locations such as the cell membrane and nucleus, where it performs non-classical functions under stress conditions. Under pathological states, the aberrant expression and function of GRP78 are extensively involved in the onset and progression of diverse human diseases, including cancer, neurodegenerative diseases, infectious diseases, cardiovascular diseases, inflammatory diseases and metabolic diseases, and often exhibit a dual role dependent on tissue specificity and disease stage. To date, a variety of intervention strategies have been developed, such as small-molecule modulators, antibodies and genetic intervention approaches. These strategies have demonstrated promising potential in preclinical studies, yet are confronted with challenges including insufficient specificity and delayed clinical translation. This paper systematically elucidates the structure, PTMs, biological functions and disease regulatory mechanisms of GRP78, summarizes the existing intervention strategies, and discusses the unresolved issues and future research directions in this field. Future research should focus on developing highly specific regulatory tools and integrating precision medicine strategies to advance the clinical translation and application of GRP78 as a therapeutic target.

## Introduction

The ER represents one of the most highly plastic organelles in eukaryotic cells, encompassing the synthesis, folding, modification, and trafficking of roughly one-third of all cellular proteins. Its luminal environment affords the essential conditions required for the proper maturation of proteins 1. Notably, the ER also functions as the primary intracellular calcium reservoir. It facilitates the maintenance of calcium homeostasis, lipid biosynthesis, and the modulation of redox equilibrium—with its own homeostasis acting as a critical determinant of cell viability and the fulfillment of cellular functions 2, 3. As a fundamental basis for cellular life processes, proteostasis serves as a corner stone dependent on the coordinated interplay of the ER chaperone system, quality control mechanisms, and stress response pathways—thereby combating intrinsic and extrinsic perturbations. When overload occurs in the protein folding capacity of the ER, proteostasis becomes compromised (characterized by the accumulation of unfolded proteins, calcium dyshomeostasis, and oxidative stress etc.) thereby triggering ERS and subsequently eliciting the UPR 4. As a stress-adaptive mechanism of cells, the UPR initially initiates an adaptive response to restore homeostasis through the coordinated action of the three major pathways (PERK, IRE1α, and ATF6); if stress persists, it activates the apoptotic pathway to eliminate damaged cells 5.

ER chaperones are indispensable for the normal physiological functions of the ER, with its core regulatory component being the 78 kDa glucose-regulated protein (GRP78)—alternatively designated as BiP or HSPA5. The discovery of GRP78 originated from investigations into virus-transformed cells in the 1970s 6. Initially, it was misidentified as a virus-specific protein. Subsequently, the team led by Ira Pastan 7 demonstrated that its expression is induced by glucose deprivation, and accordingly designated it as GRP78. Subsequent investigations have further revealed that, beyond glucose deprivation, multiple stimuli capable of impairing ER function—including calcium dyshomeostasis and hypoxia—can induce the expression of GRP78, which suggests a close association with protein folding homeostasis. In 1984, GRP78 was confirmed to be predominantly localized in the ER lumen and is capable of binding to immunoglobulin heavy chains, as well as assisting in their proper folding. This observation led to its designation as “immunoglobulin heavy chain-binding protein” 8. Its membership in the Hsp70 family was confirmed through gene sequencing, with its human-encoding gene identified as *HSPA5*. The ATPase domain of GRP78 is homologous to that of Hsp70, and the C-terminal KDEL sequence serves as a key determinant for ER localization, which thereby establishes GRP78 as a “core ER chaperone”. Classically, GRP78 is recognized as a prototypical ER lumen-resident protein, which is anchored within the ER through retrograde transport mediated by its KDEL sequence to exert chaperone functions 9. Nevertheless, a growing body of studies has demonstrated that under stress and pathological conditions, GRP78 can surmount its canonical localization constraints, translocate to the cell membrane, cytoplasm, mitochondria, nucleus, and extracellular space, and thereby exert non-canonical functions. For instance, activation of the nuclear localization signal (NLS) enables GRP78 to translocate into the nucleus, where it functions as a transcriptional cofactor to participate in gene regulation. Physiologically, GRP78 serves as the central hub for quality control of ER protein folding: it recognizes hydrophobic regions of unfolded proteins via its substrate-binding domain (SBD), utilizes ATPase activity (NBD) to supply energy for facilitating the proper folding of client proteins, and engages in the endoplasmic reticulum-associated degradation (ERAD) pathway to clear irreparably damaged proteins 10, 11. Its most pivotal function lies in acting as the central regulatory hub of the UPR: under homeostatic states, it associates with and represses the activity of IRE1α, PERK, and ATF6 in its monomeric form; upon ERS induction, GRP78 dissociates from these sensors, thereby triggering UPR signaling cascades. Furthermore, the low-affinity calcium-binding capacity of GRP78 can exert an indirect influence on the ER calcium storage pool and calcium-dependent chaperone activity 12. Under pathological conditions, dysregulated GRP78 expression and function represents a shared hallmark of multiple major diseases. Dysregulated GRP78 expression not only serves as a consequence of disease-related stress, but also actively contributes to disease initiation, progression, and therapeutic resistance.

Studies worldwide have established that GRP78 plays a central role in the progression of cancer, neurological disorders, and other human diseases by facilitating cell survival, proliferation, metastasis, and therapeutic resistance. The identification of its non-canonical functions has significantly expanded the understanding of its pathological relevance. Building on this, multiple targeted strategies have been developed, including small-molecule inhibitors such as HA15 and YUM70 13, 14, as well as monoclonal antibodies targeting cell surface-resident GRP78 and chimeric antigen receptor T-cell therapy 15, with some advancing to clinical evaluation. However, limitations remain in current research. Specifically, the ability to achieve highly specific targeting of GRP78 under pathological conditions—while avoiding normal tissue toxicity—remains a key challenge in translational medicine. Additionally, clinical research lags relatively behind, characterized by a lack of efficient biomarkers for screening eligible patients who may benefit; meanwhile, drug resistance issues also await resolution. Accumulating evidence indicates that it is essential to elucidate the role of GRP78 in human diseases.

In this review, we systematically analyze the structural features, PTMs, and physiological functions of GRP78. We further uncover its aberrant expression patterns and core mechanisms of action in human pathologies, while summarizing diverse interventional strategies targeting GRP78—with the aim of providing a theoretical basis for mechanistic investigations and therapeutic development of GRP78-associated diseases.

## Structure, post-translational modifications, and functions of GRP78

### Structure of GRP78

GRP78 is composed of two allosterically coupled domains, encompassing the NBD and the SBD. The NBD mediates nucleotide binding, while the SBD interacts with unfolded or misfolded proteins, and these two domains are linked by a flexible linker 16. The NBD is a globular structure consisting of four subdomains (IA, IB, IIA, IIB) and contains a deep cleft at its core that serves to bind ATP or ADP. The two large subdomains, IB and IIB, are linked via a flexible hinge 17. Crystal structure analyses have demonstrated that ATP binding induces the NBD to adopt an open conformation. This domain then transmits signals to the SBD via the linker helix, resulting in reduced substrate affinity of the SBD. Upon ATP hydrolysis into ADP, the NBD undergoes a conformational shift to the closed state. This conformational change regulates the activity of the SBD, leading to a drastic increase in its substrate affinity and thereby tightly locking substrate proteins. The NBD is responsible for regulating the ATPase activity of the Hsp70 family, and its conformational cycle is modulated by co-chaperones such as Hsp40/DnaJ as well as nucleotide exchange factors including GRP170/BAP. This regulatory process fuels the functional cycle of the entire chaperone 18. The SBD of GRP78 consists of two components: SBDβ (substrate-binding pocket) and SBDα (helical lid). SBDβ forms a hydrophobic groove that functions to recognize and bind the hydrophobic peptide segments of unfolded or partially folded substrate proteins. SBDα is a “lid” structure formed by α-helices, and it is linked to SBDβ via a flexible hinge region 19. The open/closed state of the SBDα “lid” is modulated by the nucleotide state of the NBD. Upon ATP binding, the lid opens, permitting rapid substrate binding and dissociation; upon ADP binding, the lid closes, sequestering the substrate within the binding pocket and preventing its misaggregation 20.

**Figure 1:**
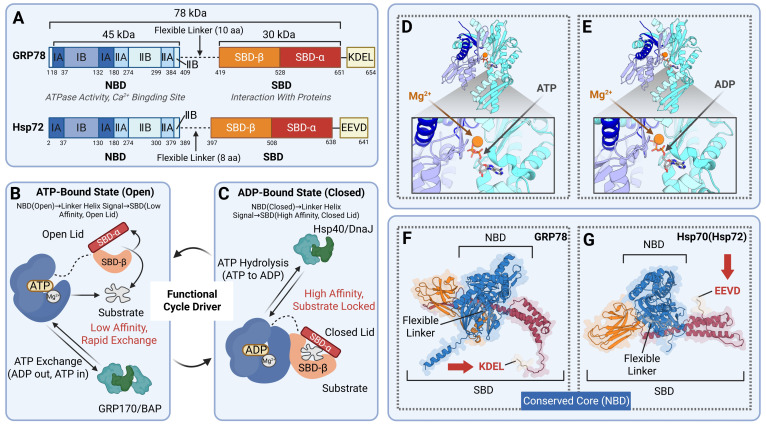
** The structure of human GRP78 and its structural differences with the representative of the Hsp70 family (Hsp72).** (A) Domains and number of amino acids of GRP78 and Hsp72. GRP78 contains a KDEL motif at its C-terminus with a long flexible linker, whereas Hsp72 has an EEVD motif at its C-terminus and a short flexible linker. (B-C) GRP78 in the ATP-bound state and ADP-bound state. When GRP78 binds to ATP, its NBD adopts an open conformation and transmits signals to the SBD via the flexible linker, keeping SBDα in an open state with low affinity for substrates, thus allowing rapid substrate exchange. When GRP78 binds to ADP, the NBD closes due to the hydrolysis of ATP to ADP, and signals are transmitted to the SBD through the flexible linker, leading to the closure of SBDα, which locks the substrate in the SBDβ pocket with high affinity for the substrate. The cofactor Hsp40/DnaJ promotes ATP hydrolysis, driving the NBD into a closed state; GRP170/BAP facilitates nucleotide exchange, pushing the NBD back to an open state. (D-E) Crystal structure of the human GRP78 ATPase domain in complex with ATP/ADP. PDB ID: (D) 3LDL; (E) 5EVZ. (F-G) Crystal structures of GRP78 and Hsp72. UniProt identifier: (F) AF-P11021-F1; (G) AF-P54652-F1.

GRP78 exhibits high homology with the Hsp70 family. As a member of the Hsp70 superfamily, GRP78 possesses an NBD domain whose core structure is highly conserved throughout the entire Hsp70 proteins. For instance, the NBD of human GRP78 exhibits more than 90% homology with the NBD of GRP78 in mice and rats. Additionally, the ATP-binding pocket, catalytic residues, and allosteric communication mechanisms are evolutionarily highly stable. While core mechanisms are conserved, GRP78 harbors distinct structural characteristics, setting it apart from other Hsp70 isoforms. Featuring an ER signal peptide at the N-terminus and a KDEL sequence at the C-terminus, it is specifically targeted to the ER, enabling it to adapt to the unique ER environment and fulfill its specialized functions. Furthermore, its SBDβ domain exhibits a higher affinity for negatively charged peptide segments, and the longer linker region between its NBD and SBD facilitates substantial interdomain mobility, thereby modulating ATPase activity and substrate binding efficiency 21. In contrast, cytoplasmic Hsp70s, such as HSPA8 and Hsp72, possess a C-terminal EEVD motif that participates in interactions with cofactors. Their SBDs show a preference for neutral hydrophobic peptides, and the linker region is relatively short 22. In terms of subcellular localization, under normal conditions, GRP78 specifically localizes to the ER, where it is involved in protein folding, quality control, and UPR signaling. Its expression is primarily induced by ERS, and its ATPase activity is activated by ER-resident DnaJ family members (e.g., ERdj4). In contrast, Hsp70 family members are distributed in compartments such as the cytoplasm and mitochondria, exhibiting broader functions. Their expression is modulated by heat shock factors (HSF), and they function cooperatively with Hsp40s (e.g., Hdj1) 23 (Table 1, Figure 1).

### Post-translational modifications of GRP78

#### Phosphorylation

As a core chaperone within the ER, the functions of GRP78 are finely regulated by both transcriptional regulation and PTMs. Among these regulatory mechanisms, phosphorylation acts as a rapid and reversible PTM, and it plays a crucial role in regulating functions of GRP78, including its ATPase activity, substrate-binding capacity, subcellular localization, and cell survival, etc. GRP78 exhibits site-specific phosphorylation, occurring primarily on Ser and Thr residues (Tyr phosphorylation has been observed in some systems), and the modification sites are mainly concentrated in the peptide-binding domain. Notably, among the three distinct functional states of GRP78—the protein-bound form, free unmodified monomer, and free modified oligomer—only the free modified oligomer undergoes phosphorylation 24. Interestingly, phosphorylation does not alter the overall conformational changes of GRP78 induced by ATP. It is specifically localized to the SBD and blocks the access of nascent substrates to the binding site through steric hindrance or charge repulsion. This renders phosphorylated GRP78 functionally inactive with an impaired capacity for substrate binding. Dephosphorylation converts GRP78 back to the monomeric state and restores its substrate binding and protein folding capabilities 25.

Specifically, Thr phosphorylation of GRP78 does not involve Thr229 in the ATP-binding domain, but is concentrated in the peptide-binding domain. The team led by Gaut 25 identified that the modified sites reside within a 47-amino-acid sequence of the peptide-binding domain, which contains seven potential Thr residues (Thr453, Thr460, Thr462, Thr473, Thr481, Thr485, Thr500). Of these residues, Thr453, Thr473, and Thr500 are highly conserved across mice, maize 26, and plasmodium 27, and serve as core modified sites. ERS can inhibit Thr phosphorylation: specifically, upon stress induction, GRP78 synthesis is upregulated, and oligomers dissociate into unmodified monomers. These monomers then undergo dephosphorylation to regain chaperone activity, which enables them to bind misfolded proteins and maintain ER homeostasis. Upon stress resolution, monomers undergo re-phosphorylation to form oligomers, returning to an inactive reserve state. Ser phosphorylation usually occurs in coordination with Thr phosphorylation and is jointly regulated by stress and kinases. In hamster fibroblasts and mouse lymphocytes, the Ser/Thr phosphorylation ratio is approximately 1:1, which collectively constitutes the primary phosphorylation modifications of GRP78 28. ERS decreases the level of Ser phosphorylation, whereas mitogen-activated protein kinase (MEK) can indirectly regulate the Ser phosphorylation of GRP78 by phosphorylating downstream proteins—such as Ser25/Ser38 of STMN1. Upon MEK activation, phosphorylated STMN1 binds to GRP78, sustaining the low Ser phosphorylation state of GRP78 and enhancing its activity, thereby increasing the migratory capacity of breast cancer (BC) cells 29. Tyr phosphorylation occurs in certain specialized systems, such as sperm, and is an unconventional modification that is relatively rare in conventional systems. In a study by the team of Vivian Lobo 30, Tyr phosphorylation of GRP78 in sperm showed dynamic changes during maturation: specifically, Tyr-phosphorylated forms of GRP78 were less abundant in immature rat testicular sperm, whereas their levels increased in mature sperm from the epididymal tail—this change was closely associated with sperm motility. In human sperm from individuals with asthenozoospermia, the level of Tyr-phosphorylated GRP78 was significantly reduced, while the unphosphorylated form increased. This observation suggests that insufficient Tyr phosphorylation may lead to sperm functional defects.

Unfortunately, current studies have not yet identified the specific sites of Ser phosphorylation and Tyr phosphorylation. However, based on the Tyr residues in the GRP78 sequence and their spatial positions, it is speculated that Tyr399 and Tyr499 may be the phosphorylation sites of GRP78. In addition, it was found that Ser64 is located near the ATP-binding pocket and belongs to a conserved “phosphorylation hot spot” region, which can be considered for experimental verification in the future. However, in previous studies, Tyr399 has been reported as a phosphorylation site of DNA methyltransferase 1 (DNMT1) 31, and neither of them is a known, common, or conserved tyrosine phosphorylation site in GRP78. Therefore, research on Ser/Tyr phosphorylation of GRP78 is still in the early stage.

#### ADP-ribosylation

Previous studies have identified that human-derived hARTC1 and hamster-derived cARTC2.1 32 can mediate the ADP-ribosylation of GRP78 33. Most members of the ARTC family are anchored via glycosylphosphatidylinositol (GPI) and localized to the extracellular side of the cell membrane. In contrast, hARTC1 is primarily localized within the ER and is capable of colocalizing with the ER marker proteins PDI and calnexin. While cARTC2.1 is a GPI-anchored protein, it can also modify GRP78 when temporarily residing in the ER 34. ADP-ribosylation of GRP78 occurs at two conserved arginine residues within the substrate-binding domain—Arg470 and Arg492 of hamster GRP78. Arg470 serves as the primary modification site, while modification of Arg492 may be dependent on Arg470. Similar to phosphorylation, ADP-ribosylation does not induce the overall structural unfolding of GRP78. This modification may disrupt the Arg470-Asp552 salt bridge and the conformation of the substrate-binding groove maintained by Arg492. The resulting disordered conformation of the substrate groove impairs the binding between GRP78 and its substrates and reduces the stability of the complex. Meanwhile, ADP-ribosylation may interfere with the allosteric coupling between the NBD and SBD, thereby mediating rapid and reversible functional inactivation regulation of GRP78 34, 35. In mouse studies, under physiological conditions, during fasting, protein synthesis in the pancreas is decreased, whereas the ADP-ribosylation level of GRP78 is enhanced. After refeeding, protein synthesis is restored while the modification level decreases. Injection of cycloheximide (a translation inhibitor) reproduces the high modification state observed during fasting 35. In the ERS response, treatment with dithiothreitol (DTT, an inhibitor of disulfide bond formation) and thapsigargin (a compound that depletes ER calcium) rapidly induces the mRNA and protein expression of hARTC1, thereby driving acute ADP-ribosylation of GRP78. Notably, this modification occurs before the stress-induced expression of GRP78 itself and occurs simultaneously with translation inhibition. Prolonged stress leads to a decrease in hARTC1 levels, which in turn causes a reduction in GRP78 modification 34, 35. Physiologically, hARTC1-mediated modification of GRP78 is an early response to ERS. When the flow of proteins into the decreases, it can temporarily sequester GRP78; after translation resumes, GRP78 is rapidly activated through de-modification, thereby preventing protein folding inhibition caused by excessive GRP78. This modification can reduce the aggregation of unfolded proteins by 40%-65% and decrease unnecessary degradation by 25.8% 35, ultimately enhancing the ability to adapt to fluctuating protein loads.

#### S-nitrosylation

S-nitrosylation, an important PTM, regulates the functions of various proteins through the transfer of NO groups to Cys residues 36. GRP78 has been identified as one of endogenous S-nitrosylated proteins. Its S-nitrosylation level is significantly reduced under high glucose conditions, potentially impairing endothelial cell function 37. Hyperglycemia, the primary pathogenic factor of diabetic vascular complications 38, induces endothelial dysfunction characterized by decreased NO bioactivity and increased superoxide production 39. Specifically, hyperglycemia reduces S-nitrosylation by promoting reactive oxygen species (ROS) generation, whereas ROS inhibitors—such as apocynin, diphenyleneiodonium, and TEMPOL—can completely reverse this reduction. Studies have demonstrated that S-nitrosylation modification inhibits the activity of protein disulfide isomerase (PDI), a central ER molecular chaperone and folding enzyme, thereby abrogating its neuroprotective function 40. Given that GRP78 also acts as an ER-resident molecular chaperone, the regulatory mechanism of its function mediated by S-nitrosylation may follow a similar pattern. Nevertheless, analysis of the GRP78 sequence via the UniProt database identifies conserved Cys residues. Considering the structural characteristics and modification preferences of S-nitrosylation, we hypothesize that Cys41 and Cys420 are potential candidate sites for this modification.

#### Ubiquitination and deubiquitination

GRP78 can undergo polyubiquitination through the ubiquitin-proteasome system and then be degraded by this system, thereby inhibiting cell migration and invasion 41. UHRF1 is a key epigenetic factor that mediates Lys48-linked polyubiquitination of GRP78, thereby promoting the degradation of GRP78. In renal tubular epithelial cells, UHRF1 modulates GRP78 via two distinct mechanisms. At the transcriptional level, it binds to the GRP78 promoter region spanning -755 to 10 bp and facilitates promoter methylation, thereby inhibiting GRP78 transcription. At the protein level, UHRF1 acts as an E3 ubiquitin ligase that directly binds to GRP78 via its SBD, mediates the Lys48-linked ubiquitination of GRP78, and promotes its proteasomal degradation. In the diabetic nephropathy (DN) model, hyperglycemia downregulates UHRF1 expression, resulting in decreased GRP78 ubiquitination and its cytoplasmic accumulation, this in turn promotes its nuclear translocation and ERS 42.

OTUD3 acts as an oncogenic factor in lung cancer, while it functions as a tumor suppressor gene in BC, and its high expression correlates with poor prognosis in patients with lung cancer 43. GRP78 serves as a specific substrate of OTUD3, and their direct interaction is dependent on the N-terminal OTU domain of OTUD3 and the C-terminal region of GRP78 (aa 500-654). This interaction displays OTU family-specificity. Specifically, the deubiquitinase OTUD3 removes the Lys48-linked ubiquitin chains from GRP78 to prolong its protein half-life and stabilize its protein levels, thereby promoting lung cancer cell proliferation, migration, and xenograft tumor growth in nude mice. Notably, knockdown of GRP78 reverses the malignant properties of lung cancer cells induced by OTUD3 overexpression 44, 45. The E3 ubiquitin ligases UHRF1 and GP78 46 mediate GRP78 ubiquitination and degradation; in contrast, OTUD3—identified as the first deubiquitinase targeting GRP78—co-regulates GRP78 protein homeostasis with these E3 ligases.

#### S-palmitoylation

Studies have demonstrated that in bladder cancer, the transcription factor SP1 transcriptionally activates the palmitoyltransferase ZDHHC9, inducing high expression of ZDHHC9 47. *ZDHHC9*—a protein-coding gene—specifically binds to GRP78 and mediates S-palmitoylation of GRP78 at the Cys420 residue. This modification enhances GRP78 protein stability and maintains its localization within the ER, thereby strengthening its inhibition of UPR sensors and ultimately contributing to bladder cancer cell proliferation, apoptosis resistance, and chemoresistance to gemcitabine and cisplatin.

#### Methylation

In 2017, Lys trimethylation of GRP78 was reported for the first time, and this modification is a key feature distinguishing its “steady-state” and “ERS-induced” subtypes 48. Studies have found that the Lys585 site of human GRP78 (corresponding to Lys586 in mice) can be trimethylated under the mediation of the methyltransferase METTL21A, forming “steady-state GRP78”. When cells experience ERS, a dynamic protein switching process is initiated: non-trimethylated ERS-induced GRP78 is robustly produced, whereas pre-existing homeostatic trimethylated GRP78 is degraded via the lysosomal pathway. This leads to a phenomenon where total GRP78 protein levels remain relatively stable in highly differentiated post-mitotic cells—including renal podocytes and the pancreatic β-cell line MIN6—with only a shift in isoform ratio. Further validation via antibody-specific assays confirmed that Lys585 trimethylation serves as a defining marker of homeostatic GRP78. Additionally, silencing of METTL21A results in the emergence of ERS-induced GRP78 even in the absence of ERS; these findings indicate that this modification suppresses the basal expression of ERS-induced GRP78. In 2020, the team led by Xu 49 demonstrated that copper exposure induces genome-wide DNA hypermethylation in the liver, including marked hypermethylation in the promoter region of GRP78. This hypermethylation exerts an effect on GRP78 expression by regulating the binding of transcription factors: specifically, CCAAT/enhancer-binding protein α is capable of binding to the methylated sequence of the GRP78 promoter, whereas C/EBPβ cannot. Consequently, this results in a significant reduction in GRP78 mRNA and protein levels.

#### S-sulfhydration

S-sulfhydration is a recently discovered PTM of proteins mediated by H_2_S, which converts the thiol group (-SH) of Cys residues into a persulfide group (-SSH). This modification acts as a key switch and regulator, altering protein enzyme activity or function, thereby regulating physiological processes including inflammation, ERS, and signal transduction 50. Notably, H_2_S-mediated protein S-sulfhydration has been shown to play an important role in various diseases. It is increasingly regarded as a major form of protein functional modification, potentially as important as phosphorylation 51. Studies have demonstrated that both endogenous H_2_S (synthesized by the cystathionine γ-lyase CTH) and exogenous H_2_S donors (e.g., NaHS) mediate S-sulfhydration of GRP78 at the Cys420 residue. This modification induces the dissociation of IRE1α from GRP78, activates the IRE1α-ERS pathway, and ultimately drives the polarization of tumor-associated macrophages (TAMs) toward the M1 phenotype—thereby inhibiting BC growth and lung metastasis 52. Notably, the Cys420 residue of GRP78 is a critical site for this modification, mutation of this site completely abrogates the aforementioned tumor suppressive effect. Furthermore, DTT can reverse this modification by releasing the -SSH group, confirming that GRP78 S-sulfhydration is an H_2_S-dependent reversible modification.

#### Acetylation

**Figure 2:**
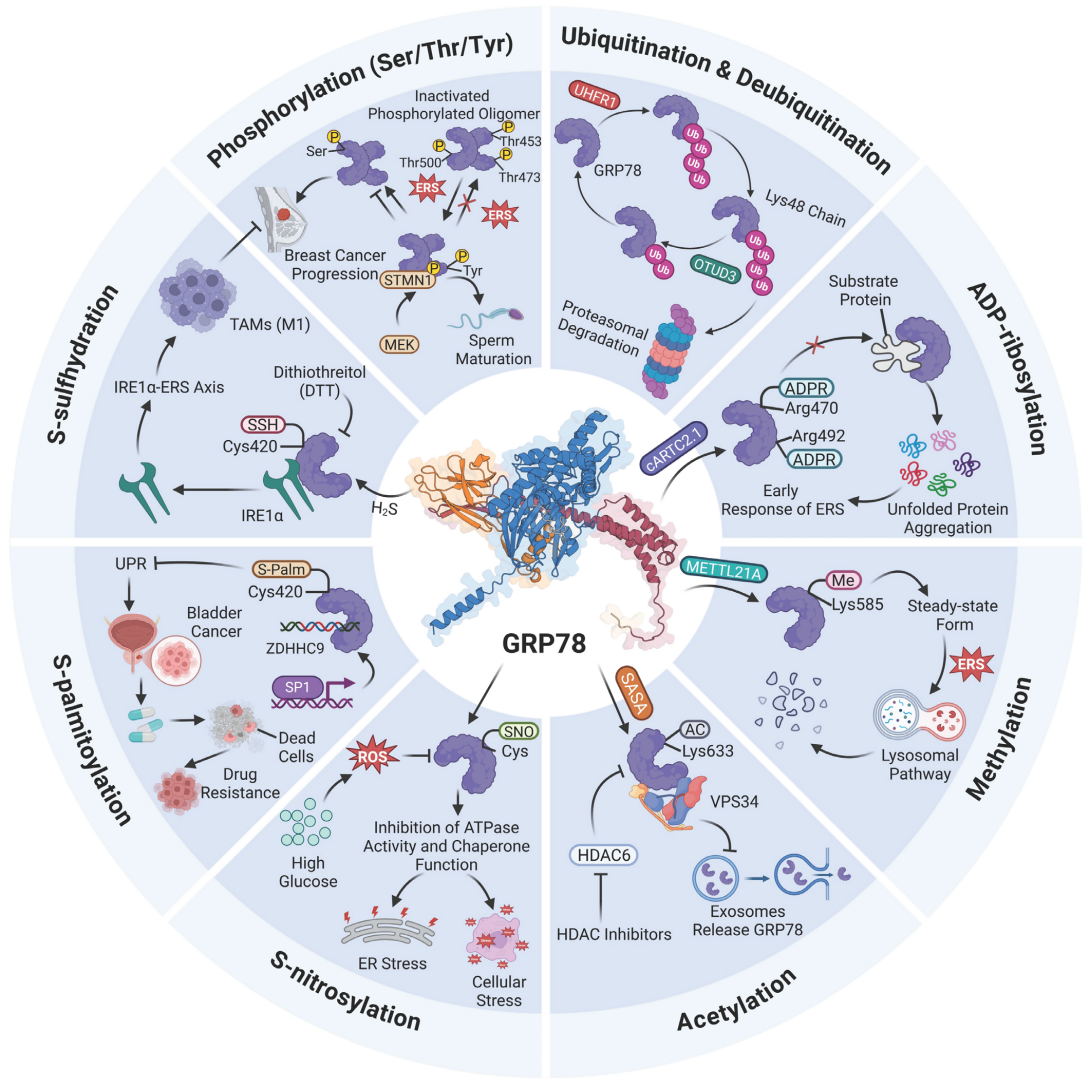
** The PTM mechanisms of GRP78.** To date, a total of eight types of PTMs have been identified on GRP78, among which the most common are phosphorylation, ubiquitination and deubiquitination, as well as acetylation. Phosphorylation of GRP78 occurs mainly on Ser/Thr/Tyr residues, and phosphorylated GRP78 exists in an inactive oligomeric state. In addition, GRP78 can be ubiquitinated by UHRF1 and subsequently degraded by the proteasome, whereas OTUD3 can deubiquitinate and stabilize GRP78. SASA catalyzes the acetylation of GRP78 at Lys633, which impairs VPS34-mediated vesicular trafficking, and this process can be regulated by HDAC6 inhibitors.

In colorectal cancer (CRC) cells, GRP78 is predominantly secreted through exosomes. Notably, histone deacetylase 6 (HDAC6)—a cytoplasmic class II deacetylase—regulates cellular functions by deacetylating non-histone proteins, including Hsp90 53 and GRP78. Its high expression is a key contributor to the low acetylation and high secretion of GRP78 in CRC cells 54. In contrast, HDAC inhibitors (e.g., SAHA, SB) induce acetylation of GRP78 at the Lys633 residue by suppressing HDAC6 activity. Acetylated GRP78 interacts with the class III phosphatidylinositol 3-kinase VPS34, impairing VPS34-mediated vesicle trafficking. This interaction prevents GRP78 from being sorted into multivesicular bodies, consequently reducing exosome release. Notably, the Lys633Q mutant—a mutant mimicking Lys633 acetylation—not only diminishes GRP78 secretion but also suppresses CRC cells growth both *in vitro* and *in vivo*. These findings confirm that GRP78 acetylation serves as a critical mechanism regulating GRP78 secretion and tumor progression 55 (Table 2, Figure 2).

Collectively, the currently identified PTMs of GRP78 include phosphorylation, ADP-ribosylation, S-nitrosylation, ubiquitination and deubiquitination, S-palmitoylation, methylation, S-sulfhydration, and acetylation. Despite distinct chemical properties, these modifications share a common core regulatory mechanism. For instance, the reversibility of these modifications serves as the foundation for their function as molecular switches. GRP78 is inactivated via phosphorylation or ADP-ribosylation under the free state, and rapidly activated through de-modification upon ERS. This process forms a dynamic cycle to promptly respond to fluctuations in the demand for protein folding. Moreover, most modifications are concentrated within the SBD of GRP78 and interfere with its binding to client proteins or downstream signaling molecules through distinct mechanisms. Nevertheless, existing studies have predominantly focused on the functional aspects, covering substrate binding, UPR signaling, protein degradation, and cellular phenotypes.

Although the functional impacts of diverse PTMs on GRP78 have been well recognized, direct and definitive experimental evidence remains lacking to clarify how these modifications induce three-dimensional conformational changes in GRP78 at the atomic level, including the allosteric interface between NBD and SBD, as well as the structural dynamics of the lid domain. While local conformation and allosteric coupling can be reasonably inferred to be modulated by PTMs via steric hindrance, electrostatic interaction, and disruption of hydrogen bonds or salt bridges based on biochemical experiments and functional alterations, high-resolution three-dimensional structures of GRP78 with PTMs at specific sites have not yet been directly resolved by cryo-electron microscopy, X-ray crystallography, or hydrogen-deuterium exchange mass spectrometry. Accordingly, how these modifications alter GRP78 function at the structural level remains elusive. Such functional changes may result from the deflection of α-helices within the SBD or the rearrangement of the allosteric coupling interface between the NBD and SBD. The precise atomic-level structural dynamics underlying these processes still require further investigation.

In addition, systematic collation of diverse PTMs of GRP78 reveals that a single amino acid residue can undergo two distinct modification patterns. For example, the Cys420 site of GRP78 is capable of undergoing two different lipid-associated modifications namely S-palmitoylation and S-sulfhydration. Since both modifications target the same thiol group, an intrinsic competitive relationship is likely to exist between them. Notably, these two modifications exhibit marked functional antagonistic effects. S-palmitoylation enhances the protein stability of GRP78 and strengthens its membrane anchoring within the ER, thereby facilitating the maintenance of ER homeostasis. In contrast, S-sulfhydration triggers the dissociation of GRP78 from IRE1α and further activates the downstream ERS pathway. We therefore speculate that cells may dynamically switch the modification pattern at the Cys420 residue by regulating the activity of upstream enzymes or modulating the local microenvironment. This enables cells to make delicate regulatory choices between maintaining cellular homeostasis for survival and initiating stress responses. Such a modification crosstalk mechanism allows GRP78 to integrate diverse upstream signals and achieve rapid functional switching at the same residue site. Further investigations into the dynamic transition between the two modifications at this site will facilitate an in-depth understanding of the multifaceted roles of GRP78 under physiological and pathological conditions.

### Biological function of GRP78

#### Maintaining ER homeostasis

As a molecular chaperone, GRP78 can bind to the hydrophobic domains of unfolded/misfolded proteins through its ATPase activity. It assists these proteins to fold correctly and recycle, thereby preventing the aggregation of unfolded protein intermediates and mediating the clearance of misfolded proteins via the ERAD pathway 56. As a low-affinity, high-capacity Ca^2+^-binding protein in the ER, GRP78 can cooperate with the ER-resident Ca^2+^-binding protein calreticulin to bind Ca^2+^, indirectly regulating Ca^2+^channel activity to maintain Ca^2+^ homeostasis of ER, thus preventing ER dysfunction caused by Ca^2+^ imbalance 57. Furthermore, GRP78 functions as a molecular regulator for the UPR, regulating the UPR pathway through interactions with ER transmembrane stress sensors. Under non-stressed conditions, GRP78 binds to PERK, IRE1α, and ATF6, maintaining them in an inactive state and thereby inhibiting UPR activation. When unfolded proteins accumulate, GRP78 is competitively dissociated, releasing PERK, IRE1, and ATF6 to trigger the three major branches of the UPR: PERK phosphorylates eIF2α, inhibits global translation to reduce ER protein load, and simultaneously selectively activates ATF4 translation; IRE1α splices XBP1 mRNA to generate the active transcription factor XBP1s, which upregulates ER foldases and ERAD components^58^; ATF6 is translocated to the Golgi apparatus where it is cleaved, and its active form enters the nucleus to upregulate ER chaperones such as GRP78 and GRP94 59. By enhancing the adaptive branch of the UPR and inhibiting its apoptotic branch, GRP78 determines cell survival or death under stress and regulates the balance of the UPR.

#### Regulating cellular autophagy

Recent studies have revealed that GRP78 is closely associated with the process of autophagy. First, GRP78 maintains ER structural stability to provide the necessary membrane basis for autophagy, and its depletion leads to ER dysfunction and autophagy blockage. In HEK293 and HeLa cells, although GRP78 knockdown can spontaneously activate the UPR pathway, it inhibits the formation of autophagosomes induced by ERS or nutrient starvation 60. The mechanisms by which GRP78 regulates autophagy include activation of signaling pathways, protein-protein interactions (PPI), epigenetic regulation and PTMs. Under stress conditions such as ERS, nutritional deprivation, or ischemia, GRP78 promotes autophagy by relieving its inhibition of PERK, leading to its phosphorylation (p-PERK). Activated p-PERK further phosphorylates eIF2α (p-eIF2α), resulting in the inhibition of global translation and selective activation of ATF4 transcription. As an autophagy regulator, ATF4 can upregulate the expression of autophagy-related genes such as LC3 61. In diabetic cardiomyopathy (DCM), melatonin reduces Vascular endothelial growth factor B (VEGF-B) levels, thereby decreasing its binding to GRP78. This causes GRP78 to dissociate from PERK, activating the PERK/eIF2α/ATF4 pathway and promoting autophagy in cardiomyocytes 62. In cancer cells, GRP78 forms a positive feedback loop with VPS34 (PI3KC3), a key autophagy kinase, thereby continuously enhancing autophagy. GRP78 overexpression inhibits miR-143 (a microRNA targeting VPS34), which relieves miR-143-mediated transcriptional repression of VPS34 and increases VPS34 expression. Meanwhile, acetylation of GRP78 can directly promote VPS34 expression, which in turn enhances LC3-II accumulation and autophagosome formation 63. In ischemic preconditioning (IPC) of neural cells, GRP78 activates autophagy via the AMPK/mTOR signaling axis. IPC induces GRP78 upregulation, which activates AMPK; as an energy-sensing kinase, AMPK can inhibit mTOR activity. mTOR acts as a negative modulator of autophagy, inhibiting its function relieves the suppression of the ULK1 complex, thereby initiating autophagy 64. In the late stage of autophagy, GRP78 inhibits autophagosome-lysosome fusion by binding to LC3 on the autophagosomal membrane, thus preventing excessive autophagy from damaging cellular structures.

#### Regulating cellular apoptosis

GRP78 plays a dual role in cellular apoptosis: it exerts anti-apoptotic effects under mild stress, while potentially promoting apoptosis under severe stress. Under normal or mild ERS conditions, GRP78 can exert anti-apoptotic effects by regulating the Akt survival pathway. As an apoptosis regulator, Akt is abnormally activated in various cancer and can mediate survival signals triggered by multiple receptors. GRP78 positively regulates the activity of the Akt pathway: normally expressed GRP78 is an important support for maintaining the function of the Akt pathway; when GRP78 is knocked down, Akt signaling is significantly suppressed. GRP78 maintains the activity of the Akt pathway by inhibiting the activation of protein phosphatase 2A (PP2A), a negative regulator of the Akt pathway, thereby reducing PP2A-mediated dephosphorylation of Akt. PP2A is a Ser/Thr phosphatase that can directly inhibit Akt pathway activation through dephosphorylation of Akt at Thr308 and Ser473 65. Furthermore, GRP78 can bind to PERK and inhibit its autophosphorylation, thereby reducing eIF2α phosphorylation and the activity of the ATF4-CHOP axis 66. CHOP, a pro-apoptotic transcription factor, can upregulate genes such as *Bim* and *Bax*, and GRP78 limits CHOP-mediated apoptosis through this mechanism. Under severe stress, GRP78 undergoes proteasomal degradation or conformational inactivation, resulting in the loss of its inhibitory effect on anti-apoptotic molecules. At this point, sustained activation of the Akt and PERK pathways, coupled with upregulated expression of CHOP, *Bax*, and other factors, promotes apoptosis.

#### Participating in immune regulation

In recent years, studies have revealed that GRP78 exerts multiple roles in immune regulation, including the modulation of inflammatory responses, autoimmunity, antiviral immunity, and cancer immunity. In transplantation immunity, a study on pancreatic β-cell transplantation by the team of Wang 67 demonstrated that GRP78 ameliorates allogeneic immune rejection through dual mechanisms: on one hand, the rate of Cytotoxic T Lymphocyte (CTL)-mediated necrosis in GRP78-transfected insulinoma cells (NIT-GRP78) is significantly reduced, preventing β-cells from CTL-induced lysis; on the other hand, GRP78 can inhibit the accumulation of oxygen free radicals to stabilize mitochondrial function, thereby protecting the survival of host cells. In autoimmune disorders including rheumatoid arthritis (RA), GRP78 rebalances immune homeostasis by promoting the production of anti-inflammatory cytokines including IL-10, inhibiting dendritic cell (DC) maturation, and enhancing regulatory T cell (Treg) responses. When internalized by myeloid cells (e.g., monocytes and DCs), GRP78 directly inhibits NF-κB activation, increases Indoleamine 2,3-dioxygenase expression, and promotes IL-10 secretion, thereby suppressing the secretion of pro-inflammatory cytokines including IL-1β and TNF-α 68. Experiments have demonstrated that in RA models, GRP78 treatment reduces inflammatory markers, and neutralization of IL-10 abrogates its anti-inflammatory effects, confirming that it alleviates autoimmune inflammation via the DC-Treg-IL-10 axis 69. In antiviral immunity, GRP78 inhibits viral replication by activating inflammatory signals: during enterovirus F infection, GRP78 directly binds to the viral 3D protein and complexes with components of the NF-κB pathway (e.g., CHUK/IKKα and IKBKB/IKKβ). This promotes IκBα degradation and p65 nuclear translocation, induces the secretion of inflammatory factors such as IL-6 and IL-8, and thereby inhibits viral replication 70. In hepatitis C virus (HCV) infection, GRP78 localizes to endosomes/lysosomes and colocalizes with Toll-like receptor 3 (TLR-3), maintaining the stability of phosphorylated interferon regulatory factor 3. This promotes the expression of interferon-stimulated genes (e.g., *ISG56*) and chemokines (e.g., RANTES, CXCL10), and enhances the innate immune response 71. In cancer immunity, taking cervical cancer as an example, GRP78 exerts bidirectional roles. On one hand, it upregulates miR-214 and miR-211 by activating the UPR, thereby inhibiting CHOP, ATF4, and apoptotic genes. It can also interact with E6/E7 proteins of HPV to stabilize them, promoting tumor progression and contributing to chemoresistance 72. On the other hand, it can exert anticancer effects by regulating autophagy and apoptosis. Moreover, ERS-induced upregulation of GRP78 can enhance the killing capacity of antigen-specific CD8^+^T cells against tumor cells, and its high expression is positively correlated with CD45RO^+^T cell infiltration in cervical cancer tissues, suggesting that GRP78 can regulate T cell-mediated immune surveillance 72.

Furthermore, under ERS conditions, GRP78 can translocate to the cell surface (csGRP78), where it acts as a pattern recognition receptor or co-receptor to regulate immune signals. It activates translocation through the IRE1α-SRC-ASAP1 axis, resulting in the dispersion of KDEL receptors and the escape of GRP78 to the cell surface. Subsequently, GRP78 binds to CD109 (a GPI-anchored protein), directing TGF-β receptors to caveolae for degradation, thereby inhibiting Smad2 phosphorylation and pro-inflammatory signals, and promoting cell survival and immune evasion 73.

#### Regulating cellular metabolism

GRP78 indirectly regulates cellular metabolism by influencing ER function. As a molecular chaperone, it assists in the folding of metabolism-related enzymes, ensuring the normal progression of metabolism of lipids and glucose. A study revealed that GRP78 silencing significantly increases the concentrations of essential polyunsaturated fatty acids including linolenic acid, linoleic acid, dihomo-γ-linolenic acid, and arachidonic acid) in BC cells. This phenomenon is attributed to the inhibition of mitochondrial fatty acid transport by GRP78 depletion: through downregulating the expression of carnitine palmitoyltransferase 1A (CPT1A), it reduces fatty acid entry into mitochondria, ultimately leading to decreased levels of fatty acid oxidation and intracellular fatty acid accumulation 74. Furthermore, GRP78 influences lipid synthesis pathways by regulating sterol regulatory element-binding protein 1 (SREBP1). Specifically, GRP78 knockdown significantly reduces the transcriptional level of SREBP1, thereby inhibiting the protein expression of its downstream key lipid synthesis enzymes: stearoyl-CoA desaturase 1 (SCD1) and fatty acid synthase (FASN). Meanwhile, GRP78 depletion slightly increases the total protein level of acetyl-CoA carboxylase (ACC) but decreases its phosphorylation level, resulting in enhanced ACC activity. This further inhibits CPT1A via malonyl-CoA, ultimately forming a metabolic phenotype characterized by “enhanced lipid synthesis and suppressed oxidation.” A study by Li and his team 75 revealed the mechanism by which GRP78 promotes glutamine metabolism through the β-catenin signaling pathway under glucose deprivation conditions. In CRC cells, glucose deprivation significantly upregulates GRP78 expression at both mRNA and protein levels, and this induction is independent of glutamine availability, indicating that GRP78 is a key metabolic stress-responsive protein under glucose deprivation. Overexpressed GRP78 disrupts the APC-β-catenin and E-cadherin-β-catenin complexes, leading to increased free β-catenin and activation of the c-MYC transcription factor. c-MYC upregulates the expression of the glutamine transporter SLC1A5 and glutaminase 1 (GLS1), thereby enhancing glutamine uptake and catabolism to provide cells with TCA cycle intermediates, NADPH, and GSH, which compensates for defects in glucose metabolism. This mechanism enables cell survival under nutrient stress, highlighting the bridging role of GRP78 in metabolic adaptation.

#### Regulating cellular signaling pathways

As a multifunctional receptor with multi-ligand binding capacity, csGRP78 forms complexes with various cell surface-anchored proteins to mediate multiple signaling pathways, thereby regulating malignant phenotypes of tumor cells such as proliferation, survival, and invasion. For example, activated α_2_-macroglobulin (α_2_M*) can bind to the specific N-terminal domain of csGRP78 to activate downstream signaling pathways, thereby promoting tumor cell proliferation, survival, and metabolic reprogramming 76. Furthermore, the α_2_M*/csGRP78 axis can upregulate prostate-specific antigen (PSA). After forming a complex with α_2_M*, PSA further binds to csGRP78 and enhances the invasiveness of prostate cancer (PC) cells by regulating DNA and protein synthesis 77.

Beyond directly binding to ligands, csGRP78 can also indirectly regulate Smad2/3 signaling by forming complexes with co-receptors such as Cripto and CD109: the Cripto/GRP78 complex inhibits TGF-β signaling, activates the Src/MAPK and Src/PI3K pathways, and promotes cancer stem cell properties 78; whereas the CD109/GRP78 complex directs TGF-β receptors into lipid rafts, blocks Smad2 activation, and impairs the tumor-suppressive function of TGF-β 73.

**Figure 3:**
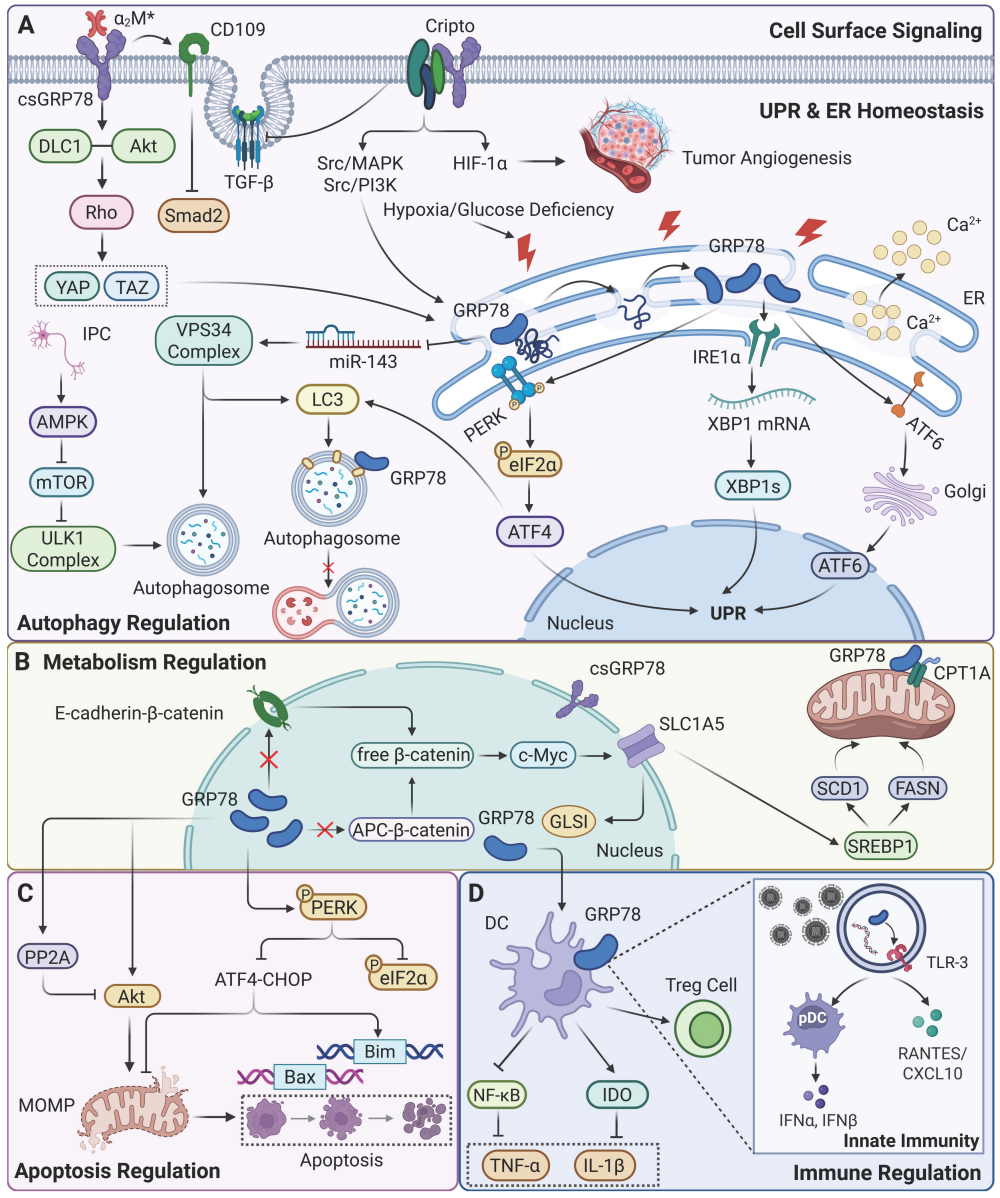
** Physiological functions regulated by GRP78.** (A) GRP78 is involved in regulating cellular signaling pathways and autophagy, maintaining UPR and ER homeostasis. (B) GRP78 is involved in regulating cellular metabolism. (C) GRP78 is involved in regulating cell apoptosis. (D) GRP78 participates in immune modulation.

In the downstream effects of signal transduction, csGRP78 further amplifies its impact on tumor phenotypes by regulating key transcription factors such as YAP/TAZ, Smad, HIF-1α, p53, c-MYC, NF-κB, and STAT3 79. For instance, radiation can upregulate csGRP78 expression, promote the formation of a complex between Akt and tumor suppressor gene *DLC1*, activate Rho signaling, and ultimately lead to increased expression and nuclear localization of YAP/TAZ. Meanwhile, the α_2_M*/csGRP78 axis can regulate the expression of YAP/TAZ target genes (e.g., *Ctgf, Cyr61, Axl*) via Rho signaling, enhancing the migratory and invasive capacities of pancreatic cancer cells 80. Furthermore, csGRP78 indirectly regulates NF-κB activation by influencing the subcellular localization of p53, thereby affecting Smad-mediated transcriptional programs 81. In hypoxic environments, csGRP78 expression is upregulated, and it regulates HIF-1α activity using Cripto as an intermediate molecule, promoting tumor angiogenesis, glucose metabolism, and invasion 82. Based on the aforementioned central role of csGRP78 in signal regulation, antibodies or peptides targeting its specific domains can interfere with downstream signaling, thereby inhibiting tumor growth and demonstrating clear therapeutic potential (Figure 3).

## The multifunctional roles of GRP78 in human diseases

ERS serves as the core defensive mechanism for cells to cope with abnormal protein folding or imbalance in calcium homeostasis, activating GRP78 via the UPR to restore ER homeostasis. As a key molecular chaperone and stress sensor within the ER, dynamic changes in the expression and function of GRP78 play a critical hub role in determining cell fate. However, numerous studies have demonstrated that sustained or excessive ERS, along with abnormal activation or dysfunction of GRP78, can induce cell apoptosis or dysfunction, thereby contributing to the occurrence and progression of various human diseases, including cancer, neurodegenerative diseases, infectious diseases, cardiovascular diseases, inflammatory diseases, and metabolic diseases. Interestingly, the biological role of GRP78 in human diseases is not static. Instead, it is subjected to multilayered regulation by expression level, PTM, subcellular localization and microenvironmental cues, thereby exhibiting strong context-dependent characteristics. Accordingly, this review begins with the expression regulation of GRP78 and analyzes its functional performance under distinct cellular contexts, while systematically comparing its specific differences across various diseases. We further summarize the context-dependent functions of GRP78 under different disease backgrounds and concludes the common regulatory principles of GRP78 in human disorders.

### Cancer

GRP78 is highly upregulated in a variety of tumors by internal and external factors including metabolic disorders and tumor microenvironment (TME) stress. By regulating key processes such as malignant transformation, metabolic reprogramming and the maintenance of stem cell properties, it endows various cancer cells with proliferative advantages, anti-apoptotic capacity as well as invasive and metastatic potential, and meanwhile mediates therapeutic resistance and is closely associated with poor prognosis 79. Moreover, csGRP78 can act as a signaling receptor to activate multiple tumorigenic pathways, exerting conserved regulatory effects in different tumor types.

#### Solid tumors

Studies have demonstrated that GRP78 is markedly upregulated in most solid tumors, and its expression level is closely correlated with tumor differentiation grade, clinical stage, and reduced overall survival. Moreover, GRP78 serves as a relevant biomarker in PC, CRC, and pancreatic ductal adenocarcinoma (PDAC) 83. One of the core mechanisms by which GRP78 drives the malignant progression of tumors is the activation of specific signaling pathways; in different solid tumors, GRP78 can regulate tumor proliferation, metastasis, and epithelial-mesenchymal transition (EMT) via the activation of distinct signaling cascades. The PI3K/Akt pathway is one of the core signaling cascades through which GRP78 regulates tumor proliferation and invasion, exerting oncogenic effects in a variety of cancers via this mechanism. In PC, csGRP78 acts as a receptor to bind ligands including α_2_M and tumor differentiation factors, thereby activating pro-survival signaling cascades including PI3K/Akt and promoting castration resistance and metastasis in cancer cells 84. GRP78 depletion can markedly inhibit Akt activation and abrogate tumorigenesis, a phenomenon that has been verified in tumor suppressor gene *PTEN*-deficient PC models 85. Similarly, in lung cancer, high expression of GRP78 induced by cytokines secreted by cancer-associated fibroblasts (CAFs) not only enhances the invasive capacity of cancer cells through the aforementioned signaling pathways 86, but also additionally activates the TGF-β/Smad2/3 pathway and the MEK1/2/ERK1/2 axis, thereby promoting the EMT program and increasing the migratory ability of lung cancer cells by threefold 87. Similar proliferation-promoting, anti-apoptotic and metastasis-promoting mechanisms have also been widely verified in various solid tumors such as bladder cancer 88, indicating that the downstream carcinogenic signal activation of GRP78 is a conserved pathogenic model across cancer types.

Beyond the classical PI3K/Akt signaling pathway, GRP78 also forms complexes with different proteins to activate disease-specific signaling cascades, thereby regulating the malignant phenotype of tumors. It is worth noting that in cancers such as BC and CRC, it is mainly csGRP78 that plays the role of activating signaling pathways. Take BC as an example, csGRP78 colocalizes with dermcidin (DCD) on the cell membrane, synergistically activating the Wnt/β-catenin signaling pathway and increasing the migratory capacity of BC cells by 2.1- to 2.8-fold 89. Similarly, in CRC, csGRP78 colocalizes with STAT3 on the cell membrane and forms a complex, which activates STAT3 phosphorylation and significantly promotes cancer cell proliferation and metastasis 90. The same disease-promoting mechanism is also followed in PDAC and gastric cancer (GC). GRP78 binds to the α_2_M* ligand and galectin-1 (Gal-1) respectively, thereby activating the Akt/DLC1 complex in PDAC, promoting the activation of Rho GTPase, and thereby regulating the nuclear localization and transcriptional activity of the YAP/TAZ signaling axis. It simultaneously regulates the VEGF/VEGFR2 pathway in GC to participate in tumor angiogenesis, ultimately enhancing the proliferation, migration and invasion abilities of cancer cells 80, 91, 92.

At present, GRP78 has been regarded as a key molecule for maintaining the “stemness” of tumor stem cells. By regulating the characteristics of tumor stem cells and inhibiting the apoptosis of cancer cells, it maintains the survival and proliferation of tumor cells. In pancreatic cancer stem cells, GRP78 is highly upregulated and maintains low intracellular ROS levels by modulating Nrf2 transcription factor-driven oxidative stress responses, thereby preventing DNA damage and lipid peroxidation, and preserving the self-renewal capacity and carcinogenic potential of stem cells 93. In head and neck cancer (HNC), cells with high expression of csGRP78 exhibit stronger cancer stem cell properties, characterized by a significantly higher proportion of cells in the G2/M phase, increased frequencies of symmetric and asymmetric division, and upregulated expression of stem cell markers such as Nanog, Oct4, and Sox2 94. Notably, GRP78 can also enhance the viability of HNC cells by downregulating the translation repressor 4E-BP1 to promote c-MYC protein expression 95, 96. In terms of maintaining cell survival, when exposed to nutrient deprivation, hypoxia and other stresses within the TME, GRP78 activates the UPR pathway, enhances protein folding capacity, and inhibits apoptosis, thereby sustaining the survival of PC cells. Interestingly, this effect can be abolished by recombinant fragment of human surfactant protein D (rfhsp-D) 84. Furthermore, in CRC, glucosidase I binds to GRP78 and recruits the deubiquitinase USP10, which removes the Lys48-linked polyubiquitin chains of GRP78 to stabilize its protein level. The stabilized GRP78 inhibits the expression of ERS-mediated CHOP and cleaved-caspase 3, thereby promoting the proliferation, migration, and invasion of CRC cells 97.

In cancer cells, GRP78 can also exert sustained oncogenic effects by sustaining its own high expression level in various ways. In both lung cancer and ovarian cancer (OC), multiple regulatory factors (such as OTUD3 or the oncogenic long non-coding RNA LINC00662) have been found to directly bind GRP78 and suppress its ubiquitination degradation, thereby prolonging the half-life of the GRP78 protein, driving cancer cell proliferation, invasion, and metastasis, and enhancing carcinogenic capacity by 2.5-fold 44, 98.

More importantly, GRP78 can attenuate tolerance of tumor cells to chemotherapy and radiotherapy via multiple mechanisms, thereby serving as a key regulatory factor mediating therapeutic resistance in various solid tumors. Studies have revealed that GRP78 is markedly upregulated in gemcitabine-resistant PDAC cell models; knockdown of GRP78 restores the sensitivity of cancer cells to gemcitabine and induces the expression of apoptosis-related genes 99. A similar mechanism has been observed in estrogen receptor-positive BC. Estrogen deprivation-induced expression of BIK activates Bax and the mitochondrial apoptotic pathway. GRP78 selectively binds to BIK and blocks this cascade, thereby promoting resistance to estrogen deprivation 100. Furthermore, GRP78 can suppress UPR activation via S-palmitoylation, or inhibit apoptosis in cancer cells by binding to or inhibiting caspase-7, thus mediating resistance to chemotherapy and radiotherapy in multiple cancers including bladder cancer, glioblastoma (GBM), and GC 47, 92, 101.

#### Hematologic malignancies

Similar to in solid tumors, GRP78 generally exerts an oncogenic role in hematologic malignancies, consistent with its function in solid tumors, while also holding potential as a prognostic biomarker. Its core functions focus on promoting tumor cell survival, maintaining stem cell properties, mediating drug resistance, and enhancing migratory and invasive capacities. In acute lymphoblastic leukemia (ALL), GRP78 overexpression facilitates the survival, migration, and infiltration of leukemia cells, preserves the properties of leukemia stem cells, and suppresses cell apoptosis. csGRP78 is highly expressed in bone marrow and peripheral blood leukemia cells of children with high-risk B-cell acute lymphoblastic leukemia. Notably, the cell cluster co-expressing csGRP78 with CXCR4, CD10, and CD19 is significantly enriched in these patients, while this cluster is absent in standard-risk patients, indicating its potential as a diagnostic stratification biomarker for high-risk ALL 102. However, interestingly, in children with T-cell acute lymphoblastic leukemia, GRP78 levels is regulated by the tumor suppressor gene *QRICH1*: low *QRICH1* expression leads to GRP78 upregulation, whereas *QRICH1* overexpression can reverse the drug-resistant phenotype by inhibiting GRP78 103.

**Figure 4:**
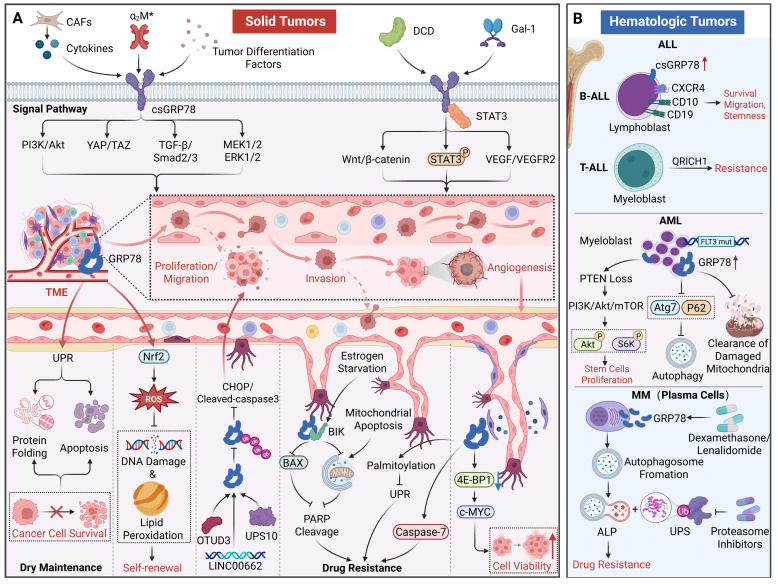
** The roles of GRP78 in cancer.** (A) In solid tumors, csGRP78 is induced by signals from the TME, thereby activating oncogenic signaling cascades including PI3K/Akt and TGF-β/Smad, as well as promoting EMT, invasion, migration, proliferation, and angiogenesis. In contrast, intracellular GRP78 maintains cancer stem cell properties and promotes cell survival and therapeutic resistance by regulating the UPR. (B) In hematologic malignancies (ALL, AML, MM), dysregulated expression of GRP78 promotes cancer stem cell proliferation, enhances cell migration, and mediates resistance to chemotherapy, targeted therapy, and proteasome inhibitors. These findings highlight that GRP78 acts as a key node in tumor progression.

Similarly, this oncogenic role of GRP78 is also prominent in myeloid leukemias. In acute myeloid leukemia (AML), GRP78 acts as a key carcinogenic driver that propels disease progression by regulating signaling pathways, mediating drug resistance, and maintaining the malignant properties of leukemia cells. Leukemia cells from both adult and pediatric AML patients exhibit marked GRP78 upregulation; moreover, GRP78 is detected on the surface of AML cell lines (e.g., KG1a, MOLM13) and primary leukemia cells, whereas its expression is barely detectable in normal hematopoietic progenitor cells and T cells 104. GRP78 expression is more pronounced in AML cells harboring FLT3 mutations (e.g., FLT3-ITD/TKD), and its expression level exhibits a negative correlation with sensitivity to FLT3 inhibitors 105. GRP78 serves as a core effector of PTEN deletion-mediated leukemogenesis: PTEN deletion activates the PI3K/Akt/mTOR pathway, enhancing the phosphorylation of Akt (Ser473/Thr308) and ribosomal protein S6 kinase, which in turn promotes the proliferation of leukemia stem cells. Furthermore, GRP78 participates in modulating mitochondrial function and autophagy; inhibition of GRP78 can promote the clearance of damaged mitochondria by regulating autophagy-related molecules such as Atg7 and P62, thereby enhancing the therapeutic sensitivity of AML cells 106. In multiple myeloma (MM), GRP78 promotes autophagosome formation to compensate for the protein degradation pathway blocked by proteasome inhibitors (e.g., bortezomib), leading to cellular drug resistance. Notably, pretreatment with dexamethasone or lenalidomide can further upregulate the expression of csGRP78, thereby establishing a vicious cycle in which drug resistance leads to elevated GRP78, which in turn drives stronger resistance 107 (Table 3, Figure 4).

It can be seen that GRP78 exhibits universal high expression, multiple oncogenic promotion, and drug resistance driving functional characteristics in human tumors. Its functions are highly dependent on its subcellular localization, PTMs and interaction networks. However, at present, most studies still take the total protein level or mRNA expression as the main analytical indicators, and the functional differentiation of different subtypes such as cell membrane localization and nuclear localization of GRP78 is not yet sufficient. In future studies, subcellular component separation combined with Tandem Mass Tags (TMT) can be considered to clarify the subtype distribution of GRP78 in different tumors. Alternatively, techniques such as immunofluorescence co-localization and multiplex immunohistochemical can be utilized to clarify the clinical significance of GRP78 at different locations. Furthermore, current research on GRP78 is highly focused on common tumor types, while systematic comparisons of rare solid tumors or different blood subtypes remain insufficient. Moreover, most of the evidence comes from *in vitro* cell lines or immunodeficient mouse models, lacking functional validation based on patient-derived xenograft models (PDX) or clinical cohort, which to some extent limits the extrapolation of the conclusion and its clinical guiding value. Future studies can integrate public databases including TCGA, GTEx, and CCLE for pan-cancer analyses, establish PDX/humanized mouse models, and validate findings in clinical cohorts, thereby enhancing the clinical translational value of GRP78 research.

### Neurological diseases

Neurodegenerative diseases are characterized by the progressive loss of neuronal function in specific regions of the nervous system, ultimately leading to severe functional impairment. These disorders present in various types, such as Alzheimer’s disease (AD), Huntington’s disease (HD), Amyotrophic lateral sclerosis (ALS) and Parkinson’s disease (PD). Although they differ in pathophysiology and clinical manifestations, they share a common pathological feature: the abnormal aggregation of misfolded proteins, which triggers ERS 10. As a core ER chaperone and a key regulator of the UPR, GRP78 frequently plays a dual role in such diseases.

#### Pathological roles

In AD and HD, the sustained high expression of GRP78 fails to exert a protective effect; instead, it participates in the pathological vicious cycle. The expression level of GRP78 in the brain of AD patients is significantly increased, and it is positively correlated with abnormal tau phosphorylation and disease stage, serving as a critical marker of early ERS activation in AD 108, 109. Aβ oligomers directly upregulate GRP78, which further increases the expression of apoptosis-associated protein GADD153. This exacerbates the accumulation of misfolded proteins and triggers neuronal apoptosis, forming a vicious cycle of “protein aggregation-ERS”. Ultimately, this cascade aggravates neuronal degeneration, synaptic damage and cognitive impairment 109, 110. In HD, the presence of toxic oligomers of mutant huntingtin (mHtt) induces the accumulation of misfolded proteins in the ER, thereby triggering the UPR 111. Studies have demonstrated that GRP78 expression is elevated in the hippocampus of HD patients. It selectively activates the PERK axis (rather than IRE1α or ATF6), which in turn inhibits dendritic spine formation and immediate early gene expression. Reduction of GRP78 expression can significantly ameliorate hippocampal pathology, alleviate the loss of dendritic spines in CA1 pyramidal neurons, decrease the density of intranuclear mHtt aggregates, and reverse memory impairment 112.

It can be seen that these two diseases share a core mechanism of the vicious cycle of “protein aggregation-excessive ERS activation”. GRP78 facilitates disease progression via excessive ERS activation and enhanced misfolded protein aggregation.

#### Protective roles

In contrast to AD and HD, GRP78 exerts a protective effect by directly targeting disease-specific pathological proteins in PD and ALS. Abnormal accumulation and aggregation of α-synuclein (α-syn) in patients with PD lead to the loss of dopaminergic neurons in the substantia nigra pars compacta (SNc), a decline in striatal dopamine (DA) levels and subsequent motor dysfunction 113. GRP78 can alleviate the loss and apoptosis of dopaminergic neurons in the SNc induced by α-syn, maintain the dopaminergic level in the striatum, and reverse the behavioral defects mediated by α-syn. Local overexpression of GRP78 in the SNc mediated by recombinant adeno-associated virus (rAAV) achieves prominent neuroprotection with merely a 39% elevation in GRP78 expression, without disturbing endogenous protein homeostasis. These findings suggest that GRP78 serves as a promising therapeutic target for PD 114. In ALS, GRP78 acts as both a stress biomarker and a regulatory therapeutic target. The core pathological hallmarks of ALS include abnormal aggregation of TAR DNA-binding protein 43 (TDP-43) 115, as well as the misfolding and aggregation of mutant copper-zinc superoxide dismutase 1 (SOD1) 116. GRP78 can specifically bind to the RNA recognition motif of TDP-43, block its misfolding and aggregation, and thereby maintain neuronal protein homeostasis 117-119. In a Drosophila model of ALS, overexpression of the GRP78 homolog Hsc70.3 markedly ameliorates TDP-43-induced ocular malformations, retinal narrowing, and vacuolization without altering the total protein level of TDP-43, which directly validates its neuroprotective role 119. Prion diseases represent a class of fatal neurodegenerative disorders characterized by spongiform encephalopathy, neuronal loss, and the accumulation of pathogenic and infectious prion proteins (PrPSc) at the expense of normal cellular prion proteins (PrPC) 120. Similarly, in prion diseases, GRP78 exerts dual protective effects: on the one hand, it directly binds to normal prion proteins, preventing their misfolding into pathogenic PrPSc and promoting the degradation of the latter; on the other hand, it alleviates ERS-induced neurotoxicity by balancing the activity of the UPR pathway 121.

In depression, GRP78 exerts an antidepressant effect by maintaining ER homeostasis and promoting AMPA receptor membrane transport to enhance excitatory synaptic transmission. In patients with major depressive disorder, GRP78 expression also exhibits a compensatory upregulation in the prefrontal cortex 122 and temporal cortex 123. However, studies have shown that chronic social defeat stress induces the upregulation of lncRNA Gm2694, which can bind to GRP78 and block its interaction with IRE1α and ATF6. This leads to the sustained activation of ERS and a reduction in the surface expression of AMPA receptors, thereby triggering excitatory synaptic deficits and depressive-like behaviors; overexpression of GRP78 or inhibition of Gm2694 can reverse this effect 124.

**Figure 5:**
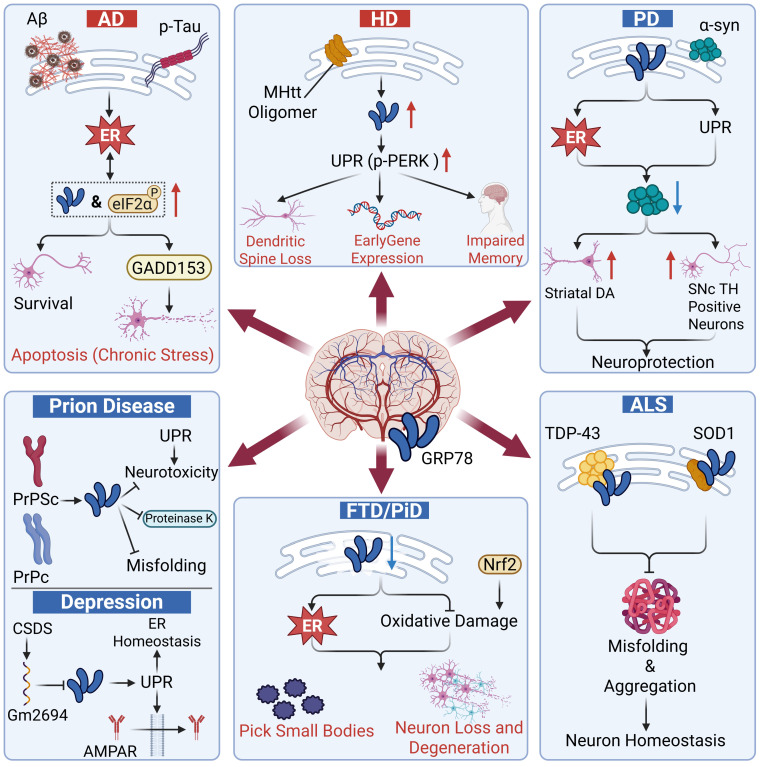
** The roles of GRP78 in neurological diseases.** In AD and HD, GRP78 primarily plays a pathogenic role. Pathogenic factors trigger ERS, leading to upregulation of GRP78, which in turn initiates the UPR, promotes chronic apoptosis, and impairs neuronal survival, resulting in memory impairment. Conversely, in PD, ALS, PiD, and depression, GRP78 exerts a neuroprotective role. It senses aberrant protein aggregation, initiates the adaptive UPR, prevents protein misfolding, and helps restore neuronal homeostasis. GRP78 plays distinct roles in different diseases, ultimately determining the fate of neurons—survival or degeneration—by mediating neuronal apoptosis, memory impairment, or neuroprotection.

Frontotemporal dementia represents a group of heterogeneous neurodegenerative disorders, among which Pick’s disease (PiD) is a rare subtype. Unlike the aforementioned diseases, the protective function of GRP78 is lost due to its own exhaustion in PiD, which is a unique abnormal stress response specific to PiD. Research has demonstrated that the level of GRP78 is significantly decreased in the cerebral cortex of PiD patients, especially in the pathologically affected areas. Consequently, GRP78 fails to cope with the accumulation of misfolded proteins, thereby exacerbating the vicious cycle of protein oxidative damage and proteasome dysfunction, disrupting the Nrf2-mediated cellular antioxidant defense and survival pathways, and ultimately contributing to the typical frontotemporal region-specific neuronal damage, tau-positive Pick body formation, and neuronal loss in PiD 125. Therefore, restoring the expression level or function of GRP78 may serve as a potential therapeutic strategy for PiD. For this purpose, we may draw on the approach mentioned earlier in PD, which uses rAAV vectors to mediate local overexpression of GRP78 to achieve neuroprotection. However, it is important to precisely control the upregulation range of GRP78 to avoid adverse reactions caused by its overexpression (Figure 5).

### Infectious diseases

Infectious diseases can be roughly divided into viral infections and bacterial infections. In such diseases, GRP78 is extensively implicated in disease pathogenesis and progression through diverse pathways, including regulating host cell responses and participating in pathogen invasion and replication.

#### Viral infection

In viral infectious diseases, on one hand, GRP78 is upregulated due to ERS caused by viral infection. It initiates the UPR, helping host cells to restore homeostasis, inhibit apoptosis, and potentially activate antiviral immune responses, thus providing cellular protection and combating viral infection. On the other hand, GRP78 may also be hijacked by various viruses: it may function as a cell surface receptor mediating viral attachment and entry, or assist in the proper folding and assembly of viral proteins during the viral replication cycle, creating favorable conditions for viral proliferation.

GRP78 mediates viral cell entry, a function that is highly conserved across diverse viruses. In COVID-19, although csGRP78 cannot mediate the binding of SARS-CoV-2 alone, it can be co-expressed with the host receptor ACE2 to form a protein complex. This complex markedly enhances the accumulation of the virus on the cell surface and its entry efficiency by directly binding to the viral spike protein 126. Similarly, GRP78 is also upregulated upon dengue virus (DENV) infection and can interact with the E protein of DENV to facilitate viral entry. Notably, silencing or cleavage of GRP78 directly abrogates the release of DENV 127. The chaperone function of GRP78 serves as a core safeguard for the maturation and assembly of viral proteins. Not only does it participate in the envelope protein maturation of Sindbis virus, HCV, vesicular stomatitis virus, and influenza A virus, among others 128, but it also provides critical support for the protein assembly and replication of a broad range of viruses. DENV, Japanese encephalitis virus, human cytomegalovirus (HCMV), Ebola virus, and hepatitis B virus (HBV) all rely on GRP78 for the assembly of their viral proteins 129. For SARS-CoV-2, GRP78 not only directly interacts with the virus’s spike protein, but also can bind to structural proteins (such as the E protein and N protein) as well as non-structural proteins (such as NSP2 and NSP14). The former are essential for maintaining viral structural integrity and infectivity, while the latter participate in the formation of the replication-transcription complex and the regulation of genome replication. In addition, through interactions with accessory protein ORF7a and ORF8, GRP78 can help the virus suppress the interferon response and evade immune surveillance 130, 131. For HBV, GRP78 is one of the most strongly induced chaperone proteins in hepatocytes. Its expression is significantly upregulated in the liver tissues of patients with chronic hepatitis B and positively correlated with the expression of p-Akt and p-mTOR. Notably, the Akt/mTOR signaling cascade can regulate the regulatory effect of GRP78 on HBV transcription and replication 132, 133. For double-stranded DNA viruses, including herpes simplex virus type 1, Kaposi’s sarcoma-associated herpesvirus (KSHV), HCMV, and vaccinia virus, GRP78 is specifically upregulated during the lytic infection phase. Notably, this upregulation in KSHV-infected cells is independent of the UPR. Notably, even when the expression of most other cellular genes is repressed, GRP78 is still markedly upregulated in KSHV-infected cells 134.

In addition, GRP78 sustains the infection process and contributes to pathological progression by regulating signaling pathways and forming positive feedback loops. ERS induced by viral infection induces the upregulation of GRP78, promoting its translocation from the ER to the cell surface. This translocation further enhances the interaction between GRP78, viral proteins, and host receptors (e.g., ACE2), leading to the generation of a self-amplifying positive feedback loop: viral infection triggers ERS, which in turn induces GRP78 upregulation and intracellular translocation, ultimately promoting viral invasion and expediting infection spread 135. Furthermore, in HBV-related pathological processes, the X protein of HBV (HBX) enhances the stability of GRP78 via E3 ubiquitin ligase TRIM25, thereby promoting its interaction with viral proteins. This subsequently upregulates the expression of *MAN1B1* gene and activates the PI3K/mTOR signaling pathway, driving the proliferation and migration of hepatocytes and ultimately correlating closely with the poor prognosis of HBV-related hepatocellular carcinoma (HCC) 133. Knockdown of GRP78 or inhibition of its activity effectively blocks the entry, replication, and infectivity of SARS-CoV-2, reducing viral load and prolonging survival in both cell cultures and mouse models 136, 137. Studies on DENV, HBV, and other viruses have also demonstrated that targeting GRP78 function directly inhibits viral replication or release, fully validating its central role in viral infections.

#### Bacterial infection

GRP78 exhibits multiple subcellular localizations in bacterial infectious diseases, including the cell surface and endosomal lumen. Through a bidirectional regulatory mechanism, it mediates host-pathogen interactions, acting not only as a facilitator of pathogenic effects by bacterial virulence factors but also as a regulatory hub in the host's anti-infection stress response.

Bacterial toxins must break through cellular barriers and complete intracellular trafficking to exert pathogenic effects. GRP78, via its unique chaperone/unfoldase activity, provides essential support for the intracellular delivery of toxins, and this mechanism is highly conserved across a variety of bacterial infections. Anthrax toxin consists of protective antigen (PA63), lethal factor (LF), and edema factor (EF). LF/EF must undergo complete unfolding to enter the cytoplasm through the PA63 endosomal membrane pore. Studies have demonstrated that GRP78 converts both LF fusion proteins and native LF from a trypsin-resistant to a trypsin-sensitive form under neutral pH conditions, and synergistically enhances toxin unfolding in an acidic environment. This unfoldase activity serves as an essential prerequisite for toxin transport through the pore formed by PA63. Knockdown of GRP78 significantly inhibits the toxic effects of anthrax toxin and suppresses cAMP elevation catalyzed by EF, indicating that GRP78 is an essential intracellular molecule for toxin action *in vivo* 138. Cholera toxin (CT), secreted by *Vibrio cholerae*, is the key pathogenic factor responsible for causing massive secretory diarrhea. After the toxin enters the lumen of the ER, GRP78, under the regulation of co-chaperone protein ERdj5, directly binds to its A subunit (CTA). ERdj5, in turn, interacts directly with Sel1L, an adaptor protein of the retrotranslocation complex Hrd1, physically anchoring the GRP78-toxin complex near the membrane transport machinery. This cascade process enables efficient translocation and retrotranslocation of the toxin from the ER lumen to the cytosol, ultimately triggering disease 139.

**Figure 6:**
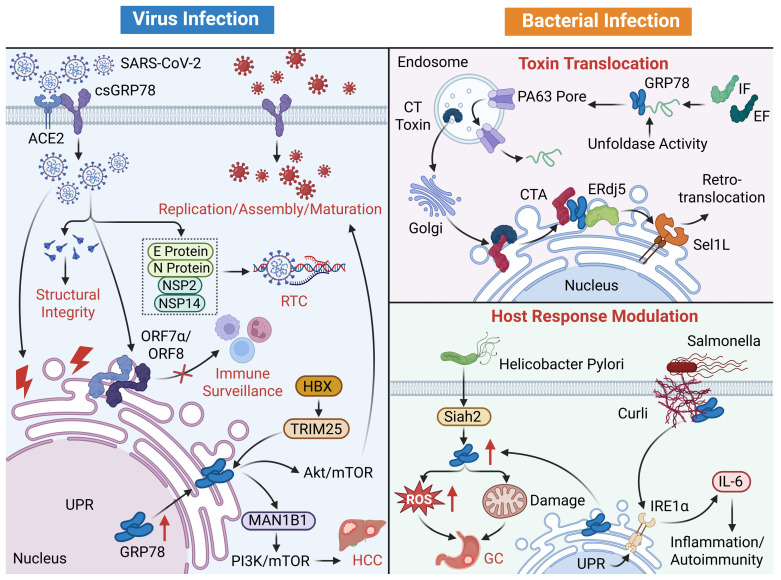
** The roles of GRP78 in infectious diseases.** In viral infections, csGRP78 assists in viral entry as well as subsequent replication, assembly, and maturation. By activating the UPR and regulating viral components, it disrupts immune homeostasis, and meanwhile connects host signaling pathways to participate in disease initiation and progression. In bacterial infections, GRP78, on the one hand, mediates the retrograde transport of bacterial toxins into the nucleus; on the other hand, it regulates related signaling pathways, inducing *Helicobacter pylori*-associated mitochondrial damage and *Salmonella*-induced inflammation and immune disorders, respectively.

In addition to directly mediating toxin translocation, GRP78 expression is regulated by bacterial infection-induced stress, and indirectly promotes disease progression by disrupting host cellular homeostasis. During *Helicobacter pylori* infection-driven gastric carcinogenesis, bacterial infection induces Ser6 phosphorylation of the E3 ubiquitin ligase Siah2, which downregulates GRP78 levels in host cells through either proteasomal degradation or secretory release. This downregulation subsequently promotes ROS accumulation, mitochondrial damage, and aggregate formation, ultimately conferring a proliferative advantage to gastric epithelial cancer cells and driving gastric carcinogenesis 140. Curli, an amyloid protein produced by *Salmonella* biofilms, can be internalized by host cells, particularly macrophages, and interacts with GRP78 in the cytoplasm, thereby activating the IRE1α branch of the UPR. This activation promotes the secretion of pro-inflammatory cytokines such as IL-6, ultimately exacerbating intestinal inflammation and autoimmune responses. This process is significantly amplified in the context of HLA-B27, a genetic risk factor for reactive arthritis (ReA) 141 (Figure 6).

### Inflammatory diseases

GRP78 plays a dual regulatory role in inflammatory diseases through the ERS-inflammation signaling axis. Its classical function can reduce the release of pro-inflammatory cytokines. However, under persistent ERS or cellular damage, GRP78 can translocate to the cell membrane or be secreted extracellularly. By binding to immune receptors such as TLR4 and CD14, it initiates downstream inflammatory cascades, thereby exacerbating inflammatory infiltration and tissue damage. In addition, GRP78 is involved in remodeling the inflammatory microenvironment by regulating the polarization balance of immune cells, including macrophages and neutrophils. Its expression levels are closely associated with the pathological progression of inflammatory diseases, such as RA and inflammatory bowel disease (IBD).

#### Anti-inflammatory effect

The protective effect of GRP78 is dominated by csGRP78, which forms a conserved anti-inflammatory regulatory pattern in intestinal inflammation and psoriasis. Both diseases achieve inflammatory remission through csGRP78-mediated regulation of immune cell function and inhibition of pro-inflammatory cytokine release, with csGRP78 levels being negatively correlated with disease activity 142, 143. In intestinal inflammation, csGRP78 expression is significantly decreased in both colitis models induced by dextran sulfate sodium (DSS) and patients with active ulcerative colitis (UC). Exogenous supplementation of csGRP78 can drive macrophages to polarize toward the M2 anti-inflammatory phenotype by suppressing the TLR4-dependent MAPK and NF-κB pathways 144, accompanied by significant upregulation of M2-associated genes including *Arg1*,* Fizz1*,* Ym1*, and *Mgl1*. Meanwhile, the administration of csGRP78 upregulated the level of tight junction proteins (claudin-4 and occludin), preventing immune cells from infiltrating the inflamed tissue—a mechanism that represents another characteristic feature of IBD 145—thus promoting the alleviation of acute colitis. In addition, upregulating the expression of csGRP78 can inhibit the secretion of pro-inflammatory mediators including TNF-α, significantly improving DSS-induced pathological manifestations, including body weight loss, colon shortening, histopathological inflammation, disease activity index, and mortality 143. However, it is worth noting that another study found that GRP78 is a key molecule promoting inflammation and apoptosis in IBD. It is overactivated in IBD, intensifying ERS and thereby leading to intestinal epithelial cell apoptosis and intestinal barrier disruption 146. Such functional switching may result from differences in subcellular localization. Extracellular csGRP78 exerts immunomodulatory and barrier-protective effects, whereas intracellular GRP78 mediates apoptosis and barrier disruption under chronic stress. In addition, disease stage may also serve as a critical influencing factor. Exogenous supplementation of csGRP78 confers therapeutic benefits during the acute phase of inflammation, while persistent stimulation under chronic inflammation enables GRP78 to drive disease progression. Nevertheless, the exact underlying mechanisms remain to be further elucidated.

In patients with psoriasis, both GRP78 expression in keratinocytes of lesional skin and serum csGRP78 levels are significantly decreased, and csGRP78 levels are negatively correlated with IL-17A levels and disease severity. Recombinant csGRP78 can directly bind to γδ T cells, inhibiting their migration and pro-inflammatory functions by downregulating the expression of CCR6 and IL-17A. However, GRP78 knockdown activates the JNK pathway, which induces the overproduction of chemokines to recruit more γδ T cells, thereby exacerbating inflammation 142.

#### Pro-inflammatory effect

In contrast, GRP78 exerts a disease-promoting role in conditions such as chronic obstructive pulmonary disease (COPD), RA and neuropsychiatric systemic lupus erythematosus (NPSLE). The core mechanism mediated by GRP78, namely activating pro-inflammatory pathways, exacerbating immune infiltration, and disrupting tissue homeostasis, is highly conserved across these disorders and positively correlated with disease activity.

**Figure 7:**
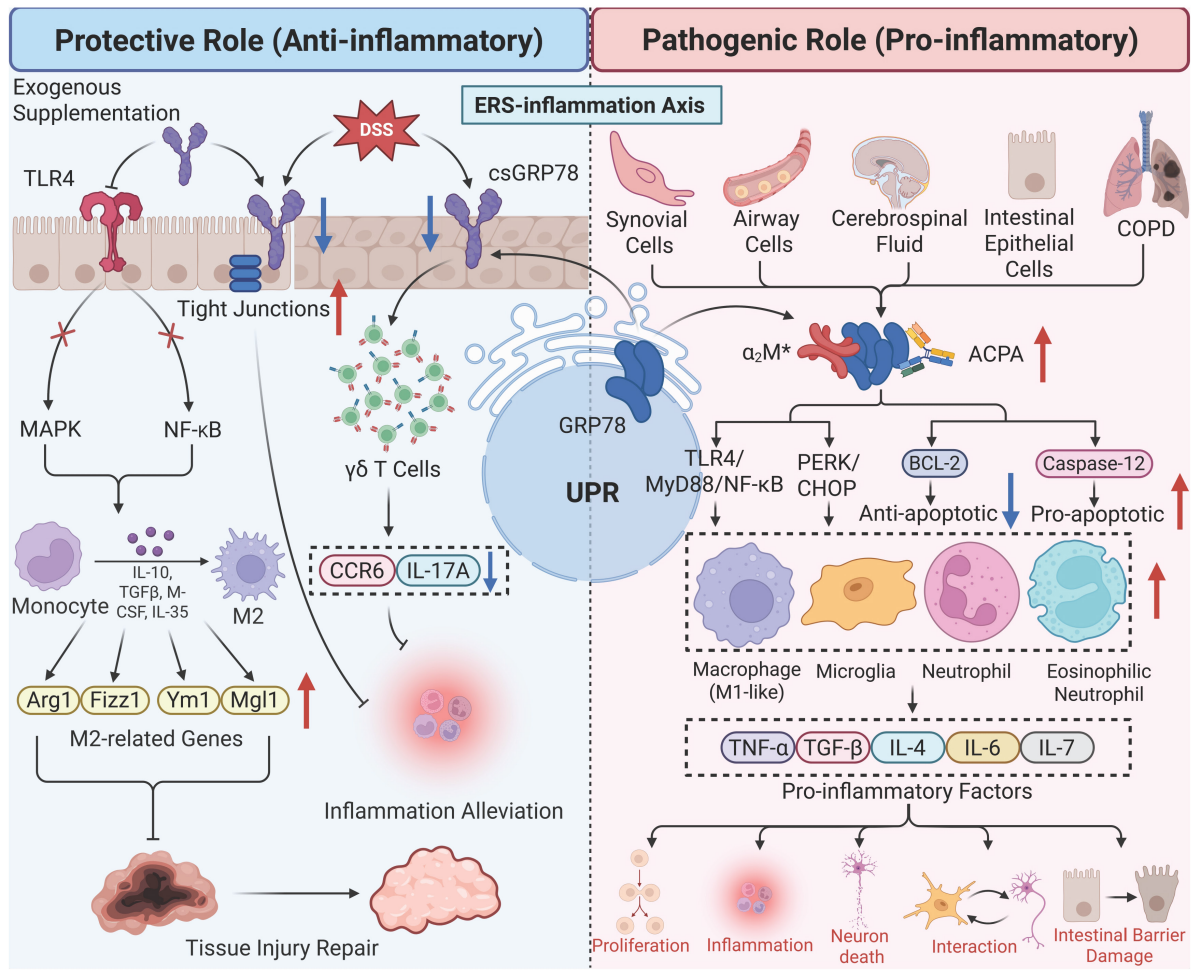
** The roles of GRP78 in inflammatory diseases.** GRP78 exerts dual functions in inflammatory diseases. Exogenous supplementation or upregulation of csGRP78 can inhibit the TLR4/NF-κB and TLR4/MAPK signaling pathways, induce M2 macrophage polarization, and suppress the migration and pro-inflammatory functions of γδ T cells, ultimately effectively alleviating inflammation and promoting tissue damage repair. However, GRP78 can also play a pathogenic role: csGRP78 mediates the activation of α_2_M*/ACPA signals, triggers the TLR4/NF-κB and PERK/CHOP pathway and the release of pro-inflammatory factors, induces the infiltration and activation of immune cells, and simultaneously regulates the apoptotic pathway to exacerbate tissue damage.

In COPD, GRP78 is significantly upregulated and participates in the inflammatory and oxidative stress processes by activating the PERK/eIF2α/ATF4/CHOP signaling pathway 147. In enterovirus 71 (EV71) encephalitis, GRP78 acts as an upstream promoter of the ERS-ferroptosis axis. EV71 virus infection induces high expression of GRP78 and CHOP, triggers ERS, leads to a decrease in glutathione peroxidase 4, a key inhibitory protein of ferroptosis, and results in iron ion accumulation, lipid peroxidation (elevated malondialdehyd), and neuronal death 148. GRP78 expression in the blood and synovial tissue of RA patients is significantly higher than that in patients with osteoarthritis (OA), and it is closely associated with disease activity (assessed by DAS28 score) and disease stage (classified by Steinbrocker classification). Mechanistically, GRP78 enhances the stress resistance of synoviocytes by inhibiting apoptotic pathways, such as upregulating BCL-2 and downregulating caspase-12, and mediates TNF-α/TGF-β-induced synoviocyte proliferation. Meanwhile, through the binding of csGRP78 to α_2_M* or anti-citrullinated protein antibodies (ACPA), it activates pro-inflammatory pathways, thereby promoting the secretion of IL-6 and TNF-α as well as angiogenesis, and exacerbating joint inflammation 149, 150. In patients with NPSLE, GRP78 is elevated in the cerebrospinal fluid. Mechanistically, GRP78 activates microglia via the TLR4/MyD88/NF-κB p65 pathway, enhancing their migratory and phagocytic capacities while promoting the release of pro-inflammatory cytokines, which disrupts neuron-microglia crosstalk 151. In patients with asthma, the expression of GRP78 is markedly upregulated in peripheral blood mononuclear cells and lung tissues of asthmatic mouse models. It exacerbates airway inflammation and elevated airway resistance by facilitating the infiltration of neutrophils and eosinophils, along with the production of pro-inflammatory cytokines including IL-4 and IL-17. Notably, GRP78 exhibits glucocorticoid insensitivity in neutrophil-dominant asthma, thereby emerging as a key mediator of refractory asthma 152 (Figure 7).

### Cardiovascular diseases

Cardiovascular diseases represent a major global health burden, characterized by complex pathological mechanisms involving endothelial dysfunction, abnormal cell proliferation, apoptosis, and ERS. As a key regulator of ERS, GRP78 has been increasingly recognized to play a dual role in the pathogenesis of various cardiovascular diseases. Its expression and functional changes are closely associated with the occurrence, progression, and severity of cardiovascular lesions, serving either as a pathogenic factor or a protective mediator depending on the disease context and microenvironment.

#### Pathological roles

GRP78 serves as a potential biomarker for atherosclerosis, and its expression level exhibits a significant positive correlation with disease severity 153. In atherosclerotic lesions, macrophages, smooth muscle cells (SMCs), and endothelial cells all overexpress GRP78, with ERS being more pronounced in unstable plaques. GRP78 exhibits a close correlation with atherosclerotic plaques within human coronary artery lesions, and it significantly enhances plaque vulnerability by mediating the apoptosis of SMCs and macrophages 154, 155. Studies have demonstrated that the small GTPase RhoA is highly expressed in the smooth muscle layer of atherosclerosis. GRP78 directly interacts with RhoA, thereby promoting the pathological proliferation, migration, invasion of vascular smooth muscle cells as well as mitophagy, which accelerates plaque formation and instability 156. In addition, during the calcification process of atherosclerosis, GRP78 and CHOP cooperatively mediate ERS-related apoptosis to promote vascular smooth muscle cell calcification; in contrast, β-hydroxybutyrate can downregulate the expression of GRP78 and CHOP via the AMPK/Nrf2 axis, inhibit the apoptotic response, and thereby reduce the aortic calcification area, calcium content, and alkaline phosphatase activity 157. Meanwhile, hemodynamic shear stress within atherosclerotic areas can modulate GRP78 levels *in vivo* as well as *in vitro*. The upregulation of GRP78 in endothelial cells is speculated to serve as a protective compensatory mechanism against ERS during early or progressive atherosclerotic lesions 158. Further studies have demonstrated that the level of anti-GRP78 autoantibodies is significantly elevated in ApoE⁻/⁻ mice (a mouse model of atherosclerosis) and patients with cardiovascular diseases, and is positively correlated with the severity of lesions. After binding to cell surface-localized csGRP78, this autoantibody can activate the NF-κB pathway, induce the production of adhesion molecules including VCAM-1 and ICAM-1 in endothelial cells, promote the adhesion of monocytes to endothelial cells, and thereby accelerate the formation of atherosclerotic lesions 159.

In cardiac hypertrophy, GRP78 also exerts a pathogenic role. By upregulating the protein expression and transcriptional activity of the transcription factor GATA4, GRP78 enhances the response of cardiomyocytes to hypertrophic stimuli such as pressure overload, thereby exacerbating myocardial hypertrophy and cardiac dysfunction. Additionally, its own expression is upregulated by hypertrophic stimuli 160. In myocardial fibrosis and T-2 toxin-induced myocardial injury, GRP78 is involved in the disease progression by activating the classic PERK-eIF2α-CHOP signaling axis. In myocardial fibrosis, GRP78 acts as an upstream activator of profibrotic signals, and its expression is significantly upregulated, which initiates this pathway to promote collagen deposition and myocardial fibrosis 161. Under the exposure of T-2 toxin, the expression of GRP78 increased sharply due to the disruption of ER homeostasis caused by T-2 toxin, activating this pathway to trigger ERS and promoting myocardial cell death and inflammation 162.

#### Protective roles

**Figure 8:**
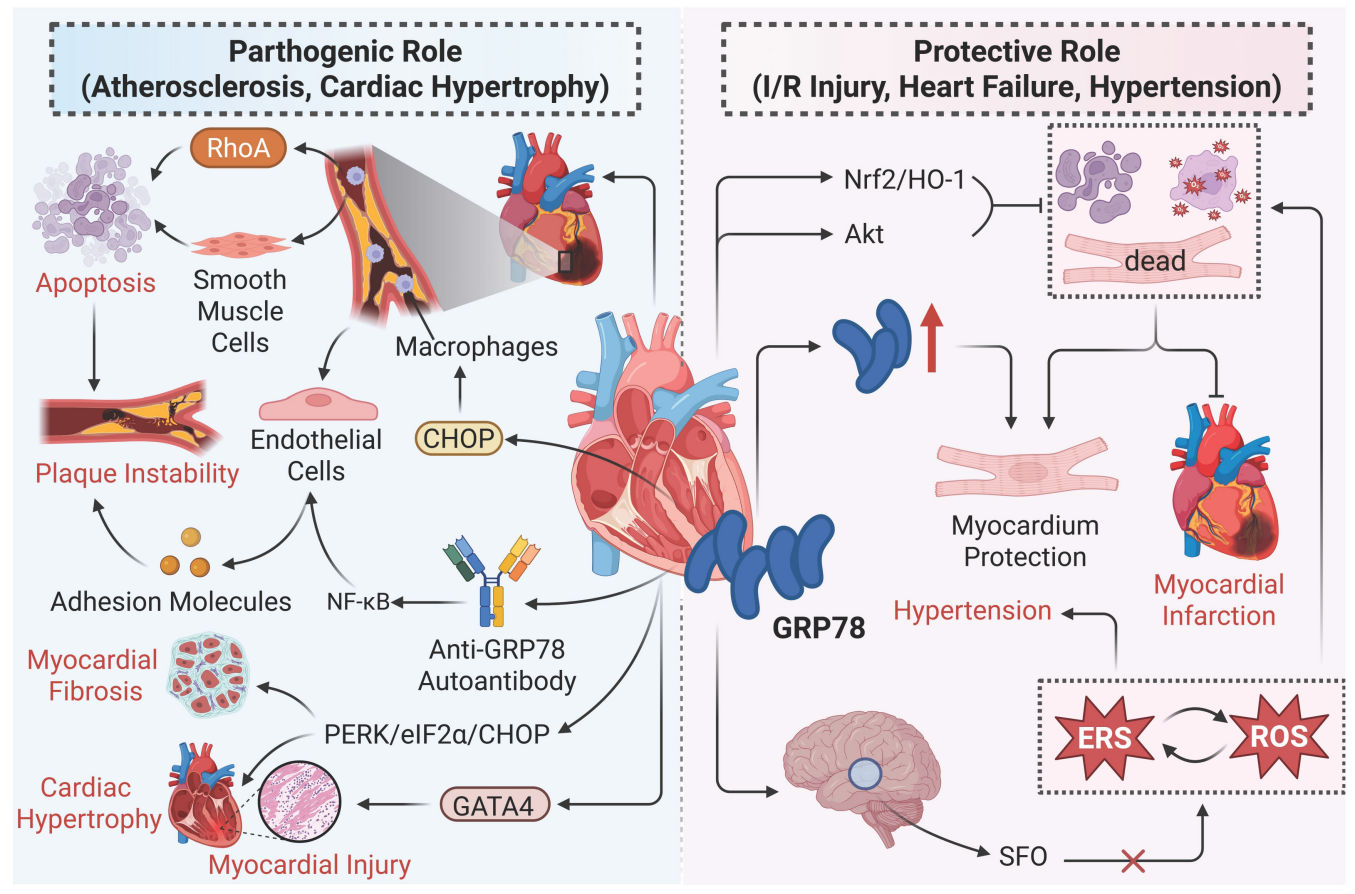
** The roles of GRP78 in cardiovascular diseases.** By activating the NF-κB and CHOP signaling pathways, GRP78 induces the apoptosis of SMCs, macrophages, and other cells, increases plaque instability, and accelerates the formation of atherosclerotic lesions. In addition, it also induces myocardial hypertrophy through the GATA4 signal and promotes myocardial fibrosis and myocardial injury by activating the PERK/CHOP signaling pathway. However, GRP78 can also achieve myocardial protection by activating related signaling pathways, and simultaneously regulate the ERS-ROS cycle and central signals to alleviate pathological injuries such as hypertension and myocardial infarction.

Interestingly, GRP78 plays a key protective role in most cases of myocardial ischemia/reperfusion injury (MI/RI). By activating anti-apoptotic and antioxidant stress-related signaling pathways (Nrf2/HO-1 axis, Akt), inhibiting ERS-mediated apoptosis, and reducing the accumulation of ROS and cardiomyocyte death, GRP78 ultimately alleviates myocardial infarction size and cardiac dysfunction 163, 164. However, there are still exceptions. A recent study has shown that under conditions of extremely severe oxidative stress induced by MI/RI, misfolded proteins accumulate in excess within the ER lumen, and GRP78 is extensively recruited to process these proteins, leading to their dissociation from IRE1α. The dissociated IRE1α is excessively activated, strongly inducing CHOP expression and triggering mitochondrial-dependent apoptosis 165. This may be attributed to the fact that the severe oxidative stress in the early stage of reperfusion results in ERS overload, whose intensity is far greater than that in the ischemic phase, thus causing GRP78 to shift from protection to injury. Additionally, compensatory elevation of GRP78 levels has been detected in patients with severe heart failure to protect cardiomyocytes 166. Similarly, in chronic hypertension, GRP78 exerts a protective role by targeting ERS in the brain’s subfornical organ (SFO), breaking the vicious cycle between ERS and oxidative stress, and inhibiting the occurrence and development of angiotensin II-dependent hypertension 167 (Figure 8).

### Metabolic diseases

Studies over the past few decades have demonstrated that patients with obesity, hyperlipidemia, fatty liver disease and diabetes mellitus (DM) exhibit abnormally elevated GRP78 expression. By regulating lipid metabolism, mitochondrial function, and the insulin signaling pathway, GRP78 plays a critical role in the progression of these metabolic diseases.

Regulation of lipid metabolism serves as a central hub for GRP78-mediated metabolic disorders. As a key adipogenic factor, GRP78, on one hand, can form a complex with the obesity-associated protein KCTD15 to orchestrate the entire process of adipocyte differentiation 168; on the other hand, acting as a lipid droplet structural protein, it activates the PPARγ and SREBP-1c/AMPK signaling pathways to promote lipid synthesis and deposition 169, 170. It is noteworthy that GRP78 exhibits tissue-specific differences in regulating lipid metabolism: in hepatic tissue, GRP78 exerts an inhibitory effect on the activation of SREBP-1c cleavage, thereby reducing lipid synthesis, whereas its deficiency can induce ectopic lipid deposition, which is an important factor in metabolic lipid disorders. At the level of energy metabolism, GRP78 suppresses mitophagy through the AMPK/mTOR axis, leading to mitochondrial dysfunction and increased accumulation of ROS. This, together with lipid metabolic disorders and insulin resistance, synergistically disrupts energy homeostasis, thereby promoting the development of metabolic diseases such as obesity, DM, and non-alcoholic fatty liver disease (NAFLD) 171, 172. Taking NAFLD as an example, GRP78 drives the progression of NAFLD from simple steatosis to non-alcoholic steatohepatitis, liver fibrosis, and even HCC by mediating ERS, regulating lipid metabolism, inducing insulin resistance, and triggering inflammatory responses 172, 173. In addition, GRP78 amplifies the metabolic disorder effect by interfering with insulin signaling and energy metabolism. As a key mediator of insulin resistance, GRP78 can activate the JNK pathway via ERS to promote IRS-1 serine phosphorylation, or downregulate Akt phosphorylation to inhibit insulin signaling, thereby reducing glucose uptake mediated by glucose transporter 4 (GLUT4) 174. More importantly, GRP78 forms a vicious cycle with hyperinsulinemia, and exacerbates metabolic imbalance through the mutual regulation mediated by transcription factor XBP-1s 126.

The biological effects of GRP78 exhibit remarkable tissue specificity. In pancreatic β-cells, its overexpression enhances ERS resistance, preserves insulin secretion function and the expression of GLUT2 on the cell surface, thereby conferring protection against obesity-associated type 2 diabetes mellitus (T2DM) and maintaining glucose homeostasis 175. In contrast, GRP78 exerts a pathogenic role in diabetic complications. In patients with DN, the expression of GRP78 is upregulated with the progression of tubular lesions, and this mechanism is associated with its nuclear translocation. Under high glucose stimulation, UHRF1 is downregulated, leading to hypomethylation of the GRP78 gene promoter (resulting in transcriptional upregulation) and reduced ubiquitin-mediated degradation of GRP78 protein. Cytoplasm-accumulated GRP78 translocates into the nucleus by binding to importin β1 via its nuclear localization signal. As a transcriptional regulator, it binds to the promoters of genes such as ATF6, XBP1, and CASP3, thereby exacerbating ERS and apoptosis in renal tubular epithelial cells 42. In DCM, the aberrant binding between GRP78 and VEGF-B disrupts the normal interaction between VEGF-B and PERK, which in turn inhibits PERK phosphorylation and its downstream pro-survival/autophagic signaling pathways, ultimately promoting myocardial pathology 62.

**Figure 9:**
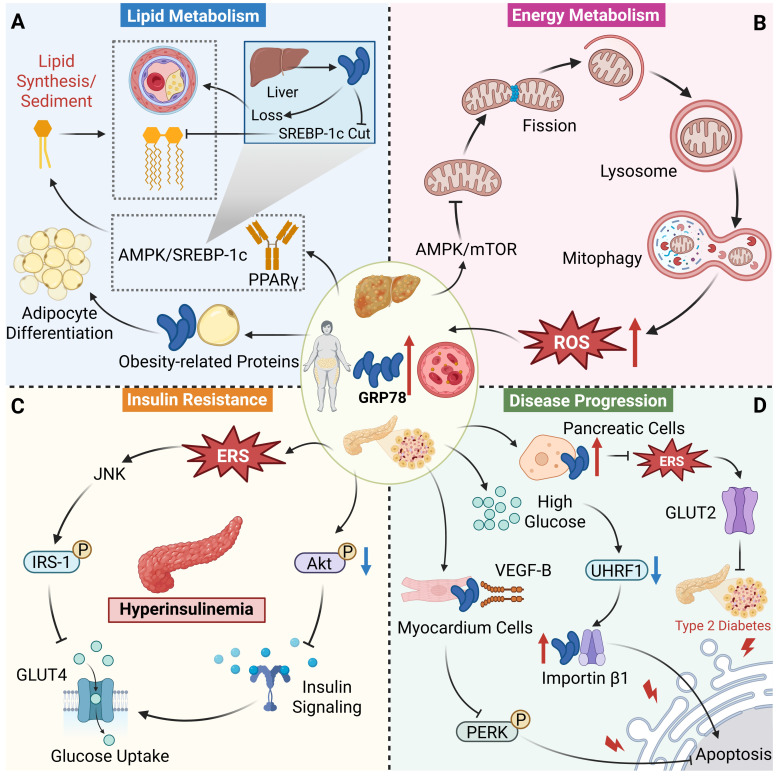
** The roles of GRP78 in metabolic diseases.** (A) In terms of lipid metabolism, GRP78 promotes adipocyte differentiation, lipid synthesis/deposition, and hepatic SREBP-1c cleavage through the AMPK/SREBP-1c/PPARγ signaling network, thereby facilitating obesity-related lipid accumulation. (B) In terms of energy metabolism, GRP78 affects mitochondrial fission and autophagy via the AMPK/mTOR pathway, exacerbating ROS production and mitochondrial dysfunction. (C) GRP78 can inhibit insulin signaling, reduce glucose uptake, and form a vicious cycle with hyperinsulinemia. (D) Sustained dysregulation of GRP78 exacerbates ERS and apoptosis of renal tubular epithelial cells, leads to cardiomyocyte damage, and ultimately promotes the pathogenesis of DN, DCM, and related cardiovascular complications.

Interestingly, the GRP78-PERK-CHOP signaling axis, which promotes fibrosis and apoptosis in cardiovascular diseases as mentioned earlier, serves as a key molecular bridge that translates exogenous injury into cellular senescence during organ aging induced by metabolic and environmental stress. Taking thymic senescence induced by short-chain chlorinated paraffins exposure as an example, environmental toxins induce oxidative stress, leading to the imbalance of ER homeostasis and a significant upregulation of GRP78 expression. By initiating the PERK-eIF2α-CHOP signaling axis, GRP78 is involved in senescence-related processes such as cell cycle arrest and cell apoptosis, ultimately resulting in functional aging of the thymus 176. In reproductive aging, postovulatory aged mouse oocytes exhibit elevated GRP78 levels, which forms a functional interaction with mitochondrial oxidative stress and serves as a marker of aging-related damage; inhibiting GRP78 can improve blastocyst development 177. From the above examples, it can be seen that the GRP78-PERK-CHOP signaling axis also follows the principle of “moderation for protection, excess for damage” in metabolic diseases (Figure 9).

The above content systematically reviews the biological functions of GRP78 in tumors, neurological diseases, infectious diseases, inflammatory diseases, cardiovascular diseases and metabolic diseases, clarifying that the core actions of GRP78 center on the regulation of ERS and the clearance of misfolded proteins, while exhibiting pronounced context-dependent roles under distinct disease conditions. The core of this dependence is reflected in the “dynamic switching of functions in response to the disease background”, and the essence of this switching lies in its mutual adaptation with the disease-specific pathological features, cellular microenvironment and regulatory network. In the field of oncology, GRP78 exhibits a “single disease-promoting” functional orientation, which is the most significant difference from other diseases. In neurological diseases, the context-dependent functional orientation of GRP78 depends on the pathological core of the disease. In infectious diseases, the functional orientation of GRP78 depends on the balance between “host defense and pathogen utilization”. In inflammatory, cardiovascular and metabolic diseases, the bidirectional function of GRP78 is highly correlated with disease stage and tissue specificity.

Nevertheless, its core mechanism remains highly conserved, and the roles of GRP78 in all diseases revolve around the core mechanism of ERS-UPR. Furthermore, the biological function of GRP78 is strongly dependent on its subcellular localization, which serves as an important molecular basis for its context-dependent effects. Intracellular GRP78 is mainly involved in ERS regulation, protein folding and transcriptional regulation. In contrast, csGRP78 mainly serves as a signaling receptor that activates downstream signaling pathways upon ligand binding. Such localization differences are commonly observed in tumors, infectious diseases and inflammatory disorders. However, current studies generally overlook the independent functions of csGRP78, nuclear GRP78, and their specific modified forms. In addition, the various limitations summarized in the section on cancers also exist in other diseases, including deficiencies in clinical validation, mechanistic interpretation, and clinical translation. Further elucidation of the isoform-specific functions of GRP78, strengthening of clinical relevance, and development of intervention strategies targeting distinct GRP78 isoforms will be essential to fully exploit the therapeutic potential of GRP78 in disease treatment.

## Multi-dimensional therapeutic strategies targeting GRP78

**Figure 10:**
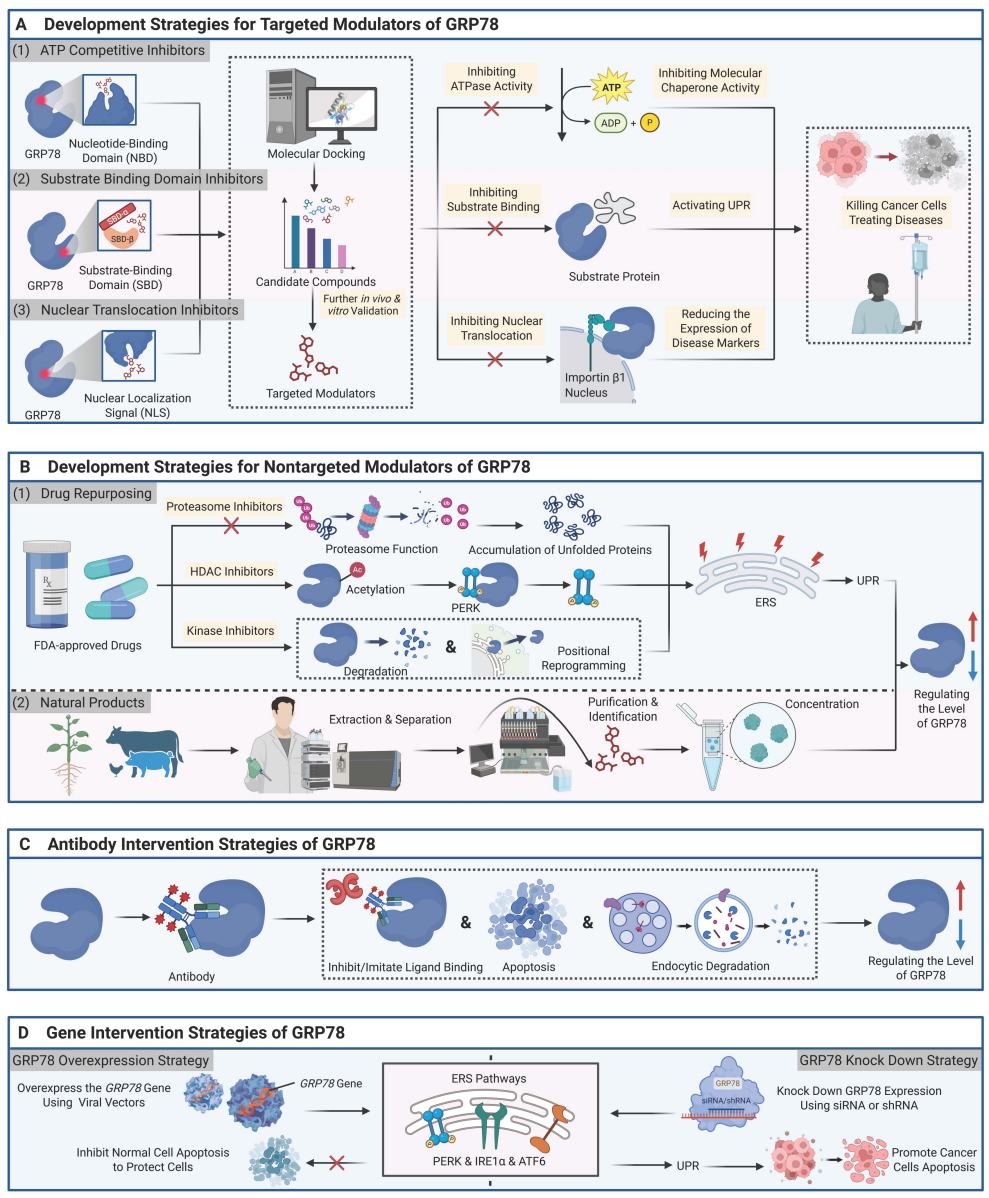
**Strategies for GRP78 modulation and intervention.** (A) The development of targeted modulators for GRP78 includes ATP-competitive inhibitors, SBD inhibitors, and nuclear translocation inhibitors, which block the ATPase activity, substrate binding, and nuclear transport functions of GRP78, respectively. (B) Non-targeted regulation of GRP78 covers drug repositioning and natural product extraction, which mainly regulate GRP78 by inducing ERS/UPR. (C) Antibody intervention against GRP78 regulates GRP78 levels by inhibiting ligand binding, inducing apoptosis, or promoting endocytic degradation. (D) Gene intervention against GRP78 achieves cell protection or promotes cancer cell apoptosis by overexpressing or knocking down GRP78.

As introduced earlier, the abnormal expression of GRP78 is strongly associated with the onset and progression of diverse human diseases. Therefore, exploring intervention strategies targeting GRP78 has become a research focus in the treatment of related diseases. At present, research on intervention strategies targeting GRP78 has been carried out at multiple levels, mainly focusing on the field of cancer. These strategies mainly include targeted regulation, non-targeted regulation, antibody intervention, and regulation at the genetic level (Figure 10).

### Targeted modulators of GRP78

Currently, research on targeted modulators against GRP78 mainly focuses on targeted inhibitors, and no targeted activators of GRP78 have been reported or developed. According to their binding sites on GRP78, these inhibitors can be broadly classified into two categories: ATP-competitive inhibitors and substrate-binding domain inhibitors. In addition, we found that another class of inhibitors exerts inhibitory effects by interfering with the NLS of GRP78, although the specific binding sites remain unclear (Figure 10A).

#### ATP-competitive inhibitors

ATP-competitive inhibitors exert their suppressive activity predominantly through targeting the NBD of GRP78. VER-155008 is a potent adenosine-derived small-molecule inhibitor that acts on the Hsp70 family. This compound competitively binds to the ATP-binding pocket of GRP78, with subsequent occupation of this locus and inhibition of ATP hydrolysis. These actions stabilize the NBD of GRP78 in a semi-open conformation, which in turn impairs allosteric signal transmission between the NBD and SBD and abrogates the chaperone function of GRP78. Experimental analysis showed that VER-155008 has a K_d_ of 0.2 μM and an IC_50_ of 2.6 ± 0.39 μM for GRP78, with a GI_50_ of 5 μM in CRC cell line HCT116 178, 179. At the cellular level, treatment with 40 μM VER-155008 elicits degradation of GRP78 client proteins in both HCT116 cells and BC cell line BT474. Co-administration with Hsp90 inhibitors exerts a synergistic effect to induce apoptosis in HCT116 cells, which is accompanied by a 70%-91.5% decrease in cellular viability 179. Additionally, (MPEG-PDLLA)-encapsulated VER-155008 nanoparticles exhibit tumor site accumulation and augment the sensitivity of CRC lesions to photothermal therapy 180. HA15 is a thiazole benzosulfonamide-derived small-molecule inhibitor with high specificity for GRP78, and it lacks cross-reactivity with other members of the Hsp70 protein family. Similarly, FRET analysis, DSC and ITC assays have demonstrated that this compound targets GRP78 at its NBD to suppress its ATPase activity, which in turn impairs the chaperone function of GRP78. These events synergistically trigger autophagy and apoptosis, ultimately inducing cancer cell death. In contrast, normal cells merely display moderate ERS with no induction of cell death. In investigations using the A375 melanoma cell line, HA15 has been shown to diminish cellular viability in a dose-dependent manner, with a IC_50_ ranging from 1 to 2.5 μM. Furthermore, this inhibitor exhibits high tumor-targeting specificity and is capable of overcoming drug resistance mediated by clinically approved agents including BRAF inhibitors (such as vemurafenib) and tyrosine kinase inhibitor (such as imatinib) 181.

PST, an endogenous peptide inhibitor of GRP78 derived from chromogranin A, engages in direct physical interaction with GRP78 in a pH-dependent fashion, exhibiting binding affinity at pH 7.4 and undergoing dissociation at pH 5.5. This interaction encompasses 66% of the amino acid sequence of GRP78. PST exerts highly specific suppression on the ATPase activity of GRP78; treatment with 1 μM or 10 μM PST results in marked inhibition of GRP78 ATPase activity, with inhibitory rates of approximately 25% and 60%, respectively, and the IC_50_ of this inhibitory effect is approximately 5.2 μM 182. While direct binding of PST to the NBD of GRP78 has not been explicitly documented in the relevant literature, functional assays including ATPase activity inhibition assays support the inference that the NBD domain constitutes the primary molecular target of PST. Notably, FL5 is a novel small-molecule inhibitor that selectively targets csGRP78. Despite binding to the NBD of GRP78, this compound does not modulate the ATPase activity of the protein. Molecular docking assays have revealed that FL5 forms merely two hydrogen bonds with the NBD of GRP78, a feature that accounts for its negligible impact on GRP78 ATPase activity. Instead, FL5 interferes with the interaction of GRP78 with downstream ligands, including pro-apoptotic factors such as Isthmin and Par-4, and disrupts the cell survival signaling pathways mediated by GRP78. Collectively, these actions confer a tumor-selective inhibitory effect that eliminates tumor cells and tumor vasculature while sparing normal cells. FL5 exhibits a relative binding efficiency of 143% to GRP78 and can elevate the T_m_ of GRP78 by 2.65 ℃. At the cellular level, FL5 has an anti-angiogenic EC_50_ of 1.514 μM against human umbilical vein endothelial cells (HUVECs). A concentration of 10 μM FL5 can induce 50% apoptosis in 786-O human renal cancer cells with high csGRP78 expression. In contrast, the compound does not exhibit cytotoxicity against normal Swiss-3T3 fibroblasts that lack csGRP78 expression 183.

In addition to the aforementioned small-molecule inhibitors, certain natural products have been proven to exert targeted inhibitory effects on the NBD of GRP78. Epigallocatechin gallate (EGCG) can downregulate GRP78 expression and UPR signaling in non-cancerous cells such as mouse retinal pigment epithelial cells 184; in cancer cells, by contrast, EGCG binds to the NBD domain of GRP78 with high affinity (K_d_ = 0.7 μM) and acts specifically on key residues including Ile61 and Glu293. This binding induces conformational changes in the NBD domain, competitively inhibits its ATPase activity, and drives its conversion from active monomers to inactive oligomers, thereby abrogating its molecular chaperone function. Furthermore, EGCG can inhibit the binding of GRP78 to caspase-7 and promote cancer cell apoptosis 185. In CRC, EGCG downregulates multidrug resistance protein 1 expression to reverse 5-fluorouracil (5-FU) resistance by inhibiting GRP78 186, and it overcomes temozolomide resistance in GBM 187. Sanguinarine can directly bind to the NBD domain of GRP78 in BC cells: it forms hydrogen bonds with Ser365, and its binding to residues including Glu293 and Arg297 generates anion-π, π-σ and hydrophobic interactions, which collectively enhance the stability of the protein-ligand complex. Similar to the previously described mechanism of action of HA15, sanguinarine inhibits the ATPase activity of GRP78 in a concentration-dependent manner (IC_50_ = 8.6 μM), abrogating its ATP hydrolysis-binding cycle and consequently leading to the loss of functional activity in protein folding and cellular homeostasis maintenance. Furthermore, it can induce the degradation of csGRP78 via the lysosomal pathway, thereby downregulating its expression and suppressing cancer cell survival 188.

#### Substrate-binding domain inhibitors

SBD inhibitors primarily exert their suppressive effects through targeted interactions with the SBD of GRP78. GRP78-IN-3 (Compound 8) is the first reported small-molecule inhibitor with specificity and subtype selectivity for the SBD of GRP78, with an IC_50_ of 0.59 ± 0.06 μM. It binds specifically to GRP78 via interactions formed between the amide bond in the molecule and the hydrophobic pocket of the SBD of GRP78 (increasing the T_m_ of GRP78 by 2.65 ℃), blocking its association with client proteins such as IRE1α. Since the chaperone function of GRP78 is persistently inhibited, the pro-survival adaptive function of the UPR is abolished, ultimately leading to cell apoptosis. Pharmacokinetic studies have shown that GRP78-IN-3 has favorable pharmacokinetic properties, including good membrane permeability, low serum binding affinity (< 10%), and strong metabolic stability 189. YUM70, a small-molecule inhibitor belonging to the hydroxyquinoline class, directly and specifically binds to the peptide-binding site within the SBD of GRP78. This compound establishes hydrogen bonds with critical amino acid residues including Ser452 and Gln458, while its quinoline ring inserts into the hydrophobic pocket constituted by Ile426, Phe451 and homologous residues. These binding interactions stabilize the conformational state of GRP78 and suppress its intrinsic ATPase activity, with an IC_50_ of 1.5 ± 0.3 μM 14. With respect to downstream signaling cascades, YUM70 upregulates the expression of 4E-BP1, which in turn abrogates eukaryotic translation initiation factor 4E-mediated translational expression of oncoproteins such as c-MYC and N-MYC, thereby potentiating anti-tumor efficacy. This mechanistic action has been experimentally validated in multiple malignancies, including BC, head and neck squamous cell carcinoma and pancreatic cancer 95. Experimental data have demonstrated that YUM70 exhibits IC_50_ values of approximately 5 μM, 7 μM and 15 μM against the pancreatic cancer cell lines MIA PaCa-2, PANC-1 and BxPC-3, respectively 14. Additionally, this inhibitor displays IC_50_ values of less than 10 μM for both N-MYC-overexpressing neuroblastoma (SK-N-BE-2) and medulloblastoma (IMR32) cell lines 95. HM03 is a small-molecule compound identified through computer-aided drug screening. This agent mimics the natural tetrapeptide substrate YZLP and directly targets the hydrophobic channel within the SBD of GRP78, which is lined by the amino acid residues Ile447, Phe472, Val482, Ile484, Ile518 and Val520. Moreover, HM03 establishes stable hydrogen bonding interactions with two key residues of GRP78: the carbonyl group of Thr463 forms a hydrogen bond with the acridine NH moiety of HM03, while the hydroxyl oxygen of Ser453 interacts with the phenolic hydroxyl group of HM03. In the HCT116 cells, treatment with 25 μM HM03 reduced cellular viability to approximately 18%, thereby conferring potent antiproliferative activity. Nevertheless, the IC_50_ value of HM03 remains undetermined to date, and no validation studies have been performed in *in vivo* animal models 190.

Furthermore, research has demonstrated that a peptide is also capable of targeting the SBD of GRP78 to exert its inhibitory function. The Bag-1 peptide is a natural peptide derived from the domain sequence of the co-chaperone Bag-1, which contains helix 1 of the C-terminal BAG domain and the N-terminal ubiquitin-like domain. This peptide directly interacts with the SBD of GRP78 (IC_50_ = 2.6 ± 0.5 μM, K_d_ = 5.7 ± 0.8 μM), abrogates its refolding activity, and triggers ERS-mediated apoptosis (e.g., cleavage of PARP and caspase-4). Studies in PC xenograft models have validated that the Bag-1 peptide exerts a potent inhibitory effect on tumor growth 191.

#### Nuclear translocation inhibitors

Nuclear translocation inhibitors exert inhibitory effects by targeting the NLS of GRP78, although the specific binding sites remain unclear. A typical example is Inauhzin-C (INZ-C), a small molecule compound with anticancer activity, which shows a direct binding affinity to GRP78 with a K_d_ of 12.73 ± 0.468 μM. The IC_50_ of INZ-C against H460 lung cancer cells is 0.37 μM. Notably, after GRP78 is knocked down, the IC_50_ of INZ-C in these cells rises to 1.10 μM, which corresponds to a threefold increase. This finding confirms the direct binding interaction between INZ-C and GRP78. Studies have demonstrated that INZ-C induces the nuclear translocation of GRP78 through the importin α/β nuclear import pathway, a phenomenon that occurs exclusively in cancer cells. Moreover, this compound inhibits cancer cell migration by downregulating vimentin, a classic mesenchymal marker, thereby exhibiting strong tumor-targeted inhibitory effects 192. Unlike INZ-C, Parishin exerts targeted inhibitory activity via direct binding to GRP78 and concomitant suppression of its nuclear translocation. This compound exhibits a K_d_ of 3.52 μM for binding to the NLS domain (residues 276–287) of GRP78, and it specifically forms hydrogen bonds with residues Lys280, Asp281 and Asn282 within this domain. These binding events abrogate the interaction between GRP78 and importin β1, thereby abrogating GRP78 nuclear translocation. In experimental models comprising high-glucose-treated renal tubular epithelial cells and db/db mice with diabetic nephropathy, Parishin markedly diminishes GRP78 nuclear translocation, downregulates the expression of ERS markers (e.g., ATF6 and XBP1) and pro-apoptotic proteins, ameliorates critical renal function parameters, and alleviates renal pathological injury and interstitial fibrosis 42 (Table 4).

Collectively, targeted modulators of GRP78 reported to date are predominantly dominated by inhibitors, as directly targeted activators remain unavailable. These inhibitors can fall into ATP-competitive inhibitors and SBD inhibitors. Both classes exert their biological activities through direct interaction with the NBD or SBD of GRP78, either suppressing its endogenous ATPase activity or disrupting its interaction with client proteins. Additionally, a small subset of GRP78 inhibitors with undefined precise binding loci have been identified, which mediate their inhibitory effects through the disruption of GRP78 nuclear translocation. However, specific activators targeting GRP78 are still unavailable; the only reported activator, BIX, upregulates GRP78 expression indirectly through the upstream ATF6 193. Future studies are required to further explore the functional pocket sites that activate GRP78, so as to provide more definite target support for the research and development of relevant modulators.

Furthermore, it can be seen that various targeted inhibitors have demonstrated excellent targeting and anti-tumor activity in *in vitro* experiments. However, except for HA15 194, EGCG 195 and Bag-1 peptide, all other inhibitors remain at the level of *in vitro* cell verification, lacking systematic verification in *in vivo* animal models. More importantly, clinical data are completely absent. Currently, no targeted inhibitor of GRP78 has entered the clinical stage; Only BOLD-100 196 has entered Phase II clinical trials (NCT04421820), which does not focus on the relevant targeting mechanisms. Thus, it is impossible to verify the safety, efficacy, and dose tolerance of such inhibitors in humans. This reveals a substantial translational gap from preclinical models to clinical practice. Regarding the obstacles to clinical translation, there may be three reasons: first, except for HA15, YUM70, INZ-C, and Parishin, most inhibitors exert varying degrees of cross-inhibition on other members of the Hsp70 family, which may interfere with the stress adaptability of normal cells. Therefore, it is necessary to clarify the effects of these inhibitors on homologous proteins such as Hsp70 in normal tissues, so as to avoid the dysfunction of normal cells caused by non-specific inhibition. Structural optimization of existing inhibitors may be adopted to reduce their impacts on other members of the Hsp70 family. Second, existing research only focuses on the short-term toxicity of inhibitors to normal cells. However, long-term inhibition of GRP78 may affect normal tissues such as pancreatic β cells and plasma cells that rely on high UPR activity. At present, systematic evaluations of long-term and organ-specific toxicity are still lacking, making it impossible to define the safety boundary for clinical application. It is necessary to clarify the toxicological mechanisms of these inhibitors and establish a long-term toxicity monitoring system. Third, there remains the issue of patient stratification. The expression level and subcellular localization of GRP78 vary significantly across different tumor types and individual patients. Existing studies have not clarified the correlation between inhibitor efficacy and GRP78 expression as well as its subcellular distribution. Blind administration of such inhibitors to all tumor patients may result in ineffectiveness in some patients and even induce toxic reactions, thereby hindering the advancement of precise therapy. Nevertheless, the current dilemma lies in the lack of reliable biomarkers for clinically screening patient populations most likely to benefit from treatment. Future efforts should focus on establishing precise patient stratification strategies and identifying specific biomarkers for GRP78-targeted therapy, so as to promote the progression from precise patient screening to personalized treatment. For instance, clinically detectable biomarkers can be developed based on the expression characteristics of GRP78, such as the membrane localization of csGRP78 and nuclear translocation levels, as well as its PTM status including Ser/Thr phosphorylation and Lys585 trimethylation. Alternatively, PET imaging probes targeting csGRP78 can be designed for *in vivo* and noninvasive monitoring of target occupancy and therapeutic efficacy of drugs in tumor tissues.

We also noted that most current data are derived from *in vitro* enzyme activity assays or single cancer cell lines such as HCT116 and A375. The effects of tumor heterogeneity and tumor microenvironment on inhibitor efficacy are rarely taken into account, which may lead to discrepancies between experimental results and real clinical scenarios. It is evident that the limitations of preclinical models also constitute an important cause of poor clinical translation. Future efforts can move beyond conventional cell lines and immunodeficient mouse models, with priority given to patient-derived organoids, humanized immune system mouse models, and gene-edited large animal models. These models can better recapitulate the human tumor microenvironment, immune system crosstalk, and organ-specific toxicity, serving as clinically more relevant experimental models. In addition, it is necessary to establish a drug efficacy evaluation system that can simulate the tumor microenvironment features such as low pH and high ATP levels. Meanwhile, systematic *in vivo* pharmacokinetic and toxicological studies should be performed to supplement preclinical evidence.

### Nontargeted modulators of GRP78

Besides the above targeted regulation strategies for GRP78, recent years have witnessed substantial progress in the regulation of GRP78 function via non-targeted pathways. Currently, non-targeted intervention methods under investigation mainly include drug repurposing of the U.S. Food and Drug Administration (FDA)-approved drugs and the development and utilization of natural products. These methods achieve indirect regulation of GRP78 by acting on upstream signaling pathways of GRP78 or indirectly affecting its activity (Figure 10B).

#### FDA-approved drugs

To date, FDA has not approved any therapeutic agents that directly target GRP78 as the sole or primary molecular target. However, several FDA-approved drugs have been shown to indirectly influence GRP78 expression and function. It is important to note that GRP78 is not the main target of these drugs, and the modulation of GRP78 usually represents either part of their primary pharmacological mechanism or a secondary downstream effect caused by the drug.

Proteasome inhibitors elicit the accumulation of unfolded or misfolded proteins via direct or indirect impairment of proteasomal function, thereby triggering ERS and activating the UPR pathway, with subsequent modulation of GRP78 expression. For instance, agents including bortezomib 197, carfilzomib 198 and atazanavir 199 exert their effects by suppressing proteasomal activity, which in turn results in the accumulation of intracellular ubiquitinated proteins. This process initiates ERS and activates UPR signaling cascades, and the upregulation of GRP78 represents an adaptive response deployed by cells to preserve protein folding homeostasis. Notably, the upregulation of GRP78 initially acts as a protective cellular response that is designed to alleviate stress and promote cell survival. However, when such stress is sustained, the elevated expression of GRP78 becomes coupled with the activation of pro-apoptotic factors (e.g., CHOP), which ultimately shifts the cellular fate toward apoptotic cell death.

HDAC inhibitors exert their effects by regulating the acetylation modification of GRP78. These drugs do not simply “inhibit” or “activate” GRP78; instead, they alter its binding properties through this PTM, thereby achieving functional reprogramming. Vorinostat 200 and panobinostat 54 are two common HDAC inhibitors. Studies have shown that vorinostat can induce specific acetylation of GRP78 at Lys585, leading to the dissociation of GRP78 from PERK, which in turn activates the UPR pathway. In contrast to vorinostat, which targets broad-spectrum HDACs, panobinostat primarily exerts its effects by specifically targeting HDAC6—a dedicated deacetylase for GRP78. This agent induces pan-acetylation of GRP78 at 11 Lys residues (e.g., Lys118, Lys122, Lys123), thereby triggering a lethal UPR in tumor cells. It is noteworthy that GRP78, as a downstream target of the UPR, although upregulated by UPR activation, loses its ability to bind PERK and inhibit its activity due to acetylation. Instead, it mediates the sustained amplification of ERS signals, thereby driving tumor cells towards apoptosis and achieving therapeutic effects.

Among kinase inhibitors, different anticancer agents exhibit distinct regulatory mechanisms toward GRP78, which can be roughly categorized into two classes: “downregulation of expression” and “localization reprogramming”. Multikinase inhibitors represented by sorafenib and regorafenib fall into the first category. These agents do not interfere with GRP78 transcription; instead, they downregulate the total expression level of GRP78 by promoting its protein degradation 201. Concomitantly, sorafenib-induced ERS activates the IRE1α-JNK signaling axis, which promotes the interaction between GRP78 and transmembrane glycoprotein CD44. GRP78 subsequently undergoes fucosylation modification prior to translocation to the cell membrane, thereby forming csGRP78 that mediates pro-survival signaling 202. Crizotinib represents the second regulatory category. Rather than directly altering total GRP78 levels, it specifically promotes the translocation of GRP78 from the ER to the cell membrane via binding directly to SRC kinase and promoting its activation. This mechanism is particularly prominent in lung cancer subtypes with ALK negativity and KRAS/EGFR mutations. By altering GRP78 localization and ligand interactions, crizotinib exerts tumor growth-inhibitory effects through a paracrine mechanism 203. Notably, certain CDK4/6 inhibitors, such as abemaciclib and ribociclib, can directly bind to the NBD of GRP78 and impair its function, which has been validated by molecular docking and other research methods. *In silico* simulations have revealed that both compounds interact with key residues (e.g., Tyr39) to form stable protein-ligand complexes; additionally, ribociclib forms an extra salt bridge with Glu201 to enhance binding affinity 204. This binding is likely to competitively inhibit ATP binding or alter the conformational state of the NBD, thereby impairing the molecular chaperone function of GRP78 and exacerbating ERS in tumor cells. However, due to the lack of verification by *in vitro* and *in vivo* experiments, the specific mechanism remains unclear. Thus, such compounds cannot be defined as targeted GRP78 inhibitors at present, and further in-depth investigations are warranted to characterize their specific binding regions and functional impacts on GRP78 in subsequent studies.

#### Natural product modulators of GRP78

Among natural compounds, polyphenols represent the largest class of natural GRP78 modulators. Their regulatory mechanisms can be primarily categorized into two types: downregulation and upregulation of GRP78 expression, with activators being relatively rare and inhibitors predominating. Polyphenolic inhibitors represented by curcumin and quercetin primarily exert therapeutic effects by inhibiting GRP78 expression or disrupting its chaperone function. Curcumin interacts with key residues of GRP78 (Arg297, Ser300, Arg367) with high binding energy (-8.5 kcal/mol). Notably, the binding interface overlaps with the functional domain of GRP78, enabling curcumin to disrupt its conformational stability and biological function. In turn, this inhibits downstream NF-κB inflammatory signaling and ERS-mediated apoptotic pathways^205^-^207^. Quercetin dose-dependently inhibits ERS markers, including GRP78 and p-PERK, in renal tissues. Its mechanisms involve scavenging ROS to block oxidative stress-mediated ERS, regulating downstream pathways of the UPR, and synergistically modulating the TLR4/NF-κB and TLR4/MAPK signaling axes, thereby synergistically enhancing anti-inflammatory and anti-apoptotic effects through multiple pathways 208, 209. Furthermore, EGCG, as mentioned earlier, is also a polyphenolic compound. Yet in contrast to curcumin and quercetin, it exerts inhibitory effects by directly targeting the NBD of GRP78, a mechanism that has been elaborated in detail in the previous subsection. However, resveratrol exhibits regulatory characteristics different from the compounds mentioned above. Studies have shown that it can time-dependently activate GRP78 protein expression in neuroblastoma cells and establish an ERS–ROS positive feedback loop to enhance this activation, thereby triggering the mitochondria-mediated apoptotic pathway 210. However, the direct binding sites and structural interaction mechanisms of quercetin and resveratrol with GRP78 remain unclear and require further investigation.

Studies have revealed that certain alkaloids exhibit a prominent dual regulatory property in the modulation of GRP78, with their specific effects varying according to cell types and pathological contexts. In some cancer models, berberine stabilizes and upregulates GRP78 expression by activating ATF6 and inhibiting the ubiquitination/proteasomal degradation of GRP78, thereby inducing autophagic cell death 211. However, in CRC and intracranial aneurysms (IA), it can indirectly inhibit GRP78 expression by activating the AMPK pathway or promoting focal adhesion kinase (FAK) phosphorylation, thus suppressing tumor cell proliferation and migration, or reducing macrophage infiltration and proinflammatory cytokine release, thus preventing the formation of IAs. Notably, studies have hypothesized that berberine may upregulate GRP78 levels via direct binding to GRP78, yet the specific interaction sites remain to be further investigated 212, 213. Interestingly, studies have demonstrated that sanguinarine—the aforementioned compound that exerts inhibitory effects by targeting the NBD of GRP78—can indirectly activate GRP78 in lung adenocarcinoma cells. It triggers the ERS-UPR pathway and upregulates GRP78 expression by inducing a burst of ROS. This upregulation, however, is actually a compensatory response to cellular stress, which ultimately leads to cell apoptosis due to excessive stress 214.

Among terpenoid compounds, shikonin and celastrol exhibit diametrically opposite regulatory effects on GRP78. In cancer cells, shikonin induces ROS production and Ca^2+^ dyshomeostasis, thereby triggering robust ERS and significantly upregulating GRP78 expression. Notably, this upregulation is not a protective response but rather elicits apoptosis by hyperactivating downstream pathways such as PERK/eIF2α/CHOP and IRE1/XBP-1, synergizing with the mitochondrial apoptotic pathway 215, 216. In contrast, in isoproterenol (ISO)-induced cardiac injury models, shikonin effectively suppresses the TLR4/NF-κB inflammatory pathway, caspase-3-mediated apoptosis, and collagen deposition, while upregulating the expression of GRP78 and other ER-associated proteins, thereby alleviating heart failure 217. In contrast, celastrol exerts a direct and irreversible inhibitory effect on GRP78. Through its quinone methide moiety, it forms covalent bonds with the Cys41 residue of GRP78, with a binding energy of -8.1 kcal/mol, which dramatically reduces the chaperone activity of GRP78 by 130-fold. This binding directly interferes with GRP78-ATP binding and ATPase activity, blocks its chaperone-mediated protein folding function, and simultaneously inhibits the interactions between GRP78 and IRE1, PERK, and ATF6, thereby preventing downstream signal activation 218. In the context of tumor therapy, this inhibitory effect induces immunogenic cell death, enhances anti-tumor immunity, and exerts a synergistic effect with PD-1/PD-L1 inhibitors, thereby reversing chemotherapeutic resistance in triple negative breast cancer (TNBC) 219.

Saponin compounds, such as ginsenosides Rg1, Rg3 and notoginsenoside R1 (NGR1), exert a consistent downregulatory effect on GRP78 expression. This regulatory effect is mainly achieved by inhibiting the ERS signaling pathway, thereby reducing cell apoptosis and tissue damage. Specifically, ginsenoside Rg1 reduces the expression of GRP78, CHOP, and cleaved caspase-12 in a dose-dependent manner and alleviates diabetes-induced cardiomyopathy by inhibiting ERS-induced apoptosis 220. In contrast, NGR1 can lower the levels of ERS response proteins (including GRP78 and p-PERK) and related pro-apoptotic proteins, delaying the onset of ERS, preventing cell apoptosis, and providing cardioprotective effects against I/R injury 221. Additionally, ginsenoside Rg3 binds to GLUT1, a protein highly expressed in tumor cells 219. Meanwhile, ginsenoside Rg1 and NGR1 synergistically inhibit oxidative stress, reducing the abnormal activation of GRP78 induced by oxidative stress. Collectively, these three ginsenosides amplify the inhibitory effect on GRP78, thereby enhancing the response to tumor immunotherapy and protecting against myocardial, renal, and other tissue injuries 221, 222 (Table 5).

So far, among natural peptide products, only the Bag-1 peptide has been found to have a regulatory effect on GRP78, as introduced earlier. Given the difficulty in identifying natural peptides that regulate GRP78, researchers have attempted to design engineered peptides for targeted intervention of GRP78. For instance, the tumor-associated peptide GMBP1 targets the SBD of GRP78, thereby competitively inhibiting its interaction with the β-catenin/ABCG2 transport protein signaling axis and disrupting the GRP78-mediated multidrug resistance mechanism in tumor cells. Additionally, the artificially designed chimeric peptide BMTP78 binds to csGRP78 and enters cells via receptor-mediated endocytosis, activating the caspase cascade. Experiments in lung cancer and bone metastasis models have demonstrated that BMTP78 significantly inhibits tumor growth and prolongs survival time 223. Compared to natural peptides, engineered peptides, via rational design and artificial optimization, exhibit superior performance in stability, functional diversity, and therapeutic application scope. These advantages make them more compatible with the requirements of clinical translation, holding particularly broad prospects in GRP78-targeted disease therapy. Thus, future research may focus on the optimization of artificial peptides, while exploring new functional domains of natural peptides to provide guidance for rational artificial design.

Compared with inhibitors that directly target GRP78, non-targeted inhibitors offer an alternative strategy with greater clinical translational potential for the treatment of GRP78-related diseases. In particular, the repurposing of FDA-approved drugs can substantially shorten the research and development cycle and reduce developmental costs. Meanwhile, natural products and engineered peptides exhibit unique advantages in safety profile and targeted optimization. Nevertheless, non-targeted agents possess more complex regulatory mechanisms, showing both distinct strengths and prominent contradictions and limitations.

Firstly, the regulatory effects of non-targeted inhibitors exhibit obvious heterogeneity. As mentioned above, the regulatory efficacy of the same compound on GRP78 is markedly dependent on cell type and pathological context, and even exerts completely opposite outcomes. Such heterogeneity lacks clear regulatory rules, making it difficult to formulate unified therapeutic regimens. Berberine upregulates GRP78 expression to induce autophagic cell death in some cancer models 211, whereas it downregulates GRP78 expression to exert inhibitory effects in CRC 212 and intracranial aneurysms 213. Shikonin elevates GRP78 levels to trigger apoptosis in cancer cells 215, 216, while it upregulates GRP78 to exert protective effects in cardiac injury models 217. The emergence of such heterogeneity may be attributed to the fact that non-targeted regulation relies on the mediation of upstream signaling pathways. The activation level and crosstalk of these upstream pathways differ substantially across cell types and pathological conditions. Furthermore, compared with targeted inhibitors with well-defined binding sites and clarified mechanisms of action, the regulatory effects of many non-targeted inhibitors have still not been well explained. For instance, whether berberine regulates GRP78 through direct binding or complete dependence on upstream pathway mediation remains lack of experimental verification. Future studies should systematically explore the correlation rules between upstream signaling pathways and GRP78 regulatory effects under different cell types and pathological conditions, clarify the binding sites and interaction modes of natural products, drugs with GRP78, and fill the existing mechanistic gaps.

In addition, non-targeted inhibitors also have the obvious defect of insufficient specificity. The targets of non-targeted inhibitors are broad-spectrum, and their regulation of GRP78 is mostly a “downstream effect”, which is prone to off-target effects. Vorinostat targets a broad range of HDACs; in addition to regulating the acetylation of GRP78, it also affects the acetylation modification of other proteins, which may lead to dysfunction of normal cells. Sorafenib, regorafenib and other multi-kinase inhibitors have multiple targets, and the downregulation of GRP78 is only a part of their mechanism of action, making it difficult to avoid their toxicity to normal tissues. However, natural products exhibit even poorer specificity, and they generally suffer from low bioavailability and poor stability. To address this shortcoming, the previously mentioned strategy of structural optimization of natural products and drugs can also be adopted to enhance their target selectivity and reduce off-target effects. For the field of cancer treatment, consideration can be given to the development of tumor microenvironment-responsive nanocarriers. A GRP78-targeted peptide-modified liposome nanoparticle (TNP^GRP78pep^) designed by the Sjoerdsma team has achieved precise delivery of DM1 (a derivative of mertansine, a potent anti-microtubule drug) prodrug for the treatment of metastatic OC 224. This successful case provides a new solution for reducing system toxicity and sets a successful example for the future development of such targeted delivery systems. Furthermore, the combined use of natural products and chemotherapeutic drugs can be considered to enhance the specificity of GRP78 regulation. A 2023 study confirmed that corilagin can enhance the anti-tumor activity of 5-FU on CRC cells by downregulating GRP78 expression, which is expected to reduce the clinical dosage of 5-FU and its associated toxicity 225. The combined effect focuses the therapeutic pressure more precisely on tumor cells with high GRP78 expression, while reducing non-selective damage to normal tissues, thereby providing a feasible strategy for indirectly improving the specificity of GRP78 regulation.

### Antibody intervention strategies

C38 and C107 are both mouse monoclonal immunoglobulin G antibodies targeting the C-terminal domain of GRP78, with specific affinity for the COOH-terminus of GRP78 (K_d_ of 2.19 nmol/L and 2.67 pmol/L, respectively) 226. C38 inhibits the binding of α_2_M* to the NH2-terminus of GRP78 via steric hindrance, thereby suppressing α_2_M*-induced signaling pathways. In PDAC tumor models, C38 blocks the GRP78-mediated Rho/YAP/TAZ signaling axis, reduces the motility and invasiveness of PDAC cells, enhances radiosensitivity, and decreases the risk of tumor recurrence after radiotherapy 227. In contrast to C38, C107 directly triggers the mitochondrial apoptotic pathway without relying on antagonizing ligand binding. In melanoma cells, it upregulates the activity of the p53 tumor suppressor protein, promotes chromatin fragmentation, activates the caspase-dependent apoptotic pathway, induces tumor cell apoptosis, reduces the infiltration of immunosuppressive cells, and thereby indirectly improves the tumor immune microenvironment 226, 228. Interestingly, while N88, like C38 and C107, is a mouse monoclonal antibody targeting GRP78, it interacts with the NH_2_-terminus of GRP78 and exhibits unique agonistic activity. With relatively weak affinity for GRP78 (K_d_ = 0.12 μM), N88 mimics the function of α_2_M*, activates the PI3K/Akt signaling pathway, promotes tumor cell proliferation, and inhibits apoptosis. However, this effect can be blocked by C38 226.

PAT-SM6 is a fully human IgM monoclonal antibody that specifically targets the GRP78 variant expressed exclusively on the surface of malignant tumor cells. Via its pentameric structure, it enables multivalent binding to clustered GRP78, with a K_d_ of approximately 4 nM for GRP78 and around 20 nM for low-density lipoprotein (LDL)/oxidized LDL under this binding state. PAT-SM6 exerts its anti-tumor effects primarily by inducing lipoptosis, activating complement-dependent cytotoxicity, and blocking the GRP78-mediated autophagy-related chemoresistance pathway. When combined with lenalidomide, bortezomib or dexamethasone, it synergistically inhibits the proliferation of multiple myeloma cells 107, 229, 230. In contrast, MAb159 targets csGRP78 in its native conformation, can recognize homologous GRP78 in both humans and mice, and exhibits high affinity for human GRP78 with a K_d_ of 1.7 nM. However, both antibodies can attenuate the tumorigenic activity of csGRP78 by inducing its internalization: the former reduces the surface expression of GRP78 via the clathrin-dependent endocytic pathway, while the latter is primarily characterized by endocytosis-mediated excessive lipid uptake. Via these endocytic processes, MAb159 blocks the PI3K/Akt signaling pathway, triggers tumor cell apoptosis, inhibits angiogenesis, thus exerting growth-suppressive and metastasis-blocking effects in tumor models such as CRC 231.

In contrast to the aforementioned monoclonal antibodies, SPA-826 is a polyclonal antibody targeting GRP78. Yet its mechanism is analogous to that of C38, C107 and MAb159: it specifically recognizes the COOH-terminus of GRP78 and exerts its effects in a csGRP78 expression-dependent manner. Furthermore, SPA-826 inhibits the autophosphorylation and activation of GRP78 by antagonizing the binding of α_2_M*, thereby exhibiting prominent antiproliferative and proapoptotic activities in PC cells 1-LN 228. Meanwhile, as a polyclonal antibody, SPA-826 recognizes multiple epitopes within the C-terminal domain of GRP78, which renders it potentially endowed with broader binding characteristics and greater research value than monoclonal antibodies. Currently, SPA-826 is more commonly employed as an experimental tool for detecting GRP78 expression, and it is compatible with a variety of experimental techniques including Western Blot, immunoprecipitation, and flow cytometry. This constitutes a distinct experimental application advantage that monoclonal antibodies do not possess simultaneously (Figure 10C).

### Gene intervention strategies

Genetic engineering has demonstrated great potential in disease therapy by modulating GRP78 expression. Currently, this approach encompasses two major strategies: overexpressing the GRP78 protein to enhance its chaperone function, and knocking down GRP78 to increase the chemotherapeutic sensitivity of cancer cells.

As early as 2010, a research team delivered the *GRP78* gene via an AAV vector to achieve GRP78 overexpression, which was applied for the treatment of retinitis pigmentosa. In rat models of hereditary retinal degeneration, the AAV5 vector delivered the *GRP78* gene through subretinal injection, which downregulated ERS markers such as CHOP and caspase-7, alleviated ERS, inhibited photoreceptor cell apoptosis, and significantly restored electroretinogram amplitude as well as retinal structural integrity 232. Furthermore, Ha and his colleagues 233 utilized an AAV2 vector for intravitreal injection to overexpress GRP78 in a mouse model of optic nerve crush injury. By downregulating the ERS pathway and reducing abnormal tau protein aggregation, this strategy protected the survival of retinal ganglion cells and improved visual function.

Genetic engineering techniques, particularly RNAi technology such as siRNA or shRNA, are widely employed to knock down GRP78 expression for investigating its biological functions and developing therapeutic strategies. For instance, lentivirus-mediated shRNA was used to specifically silence GRP78 in rat pancreatic acinar cells; this intervention enhances the activation of caspases, inhibits the activity of X-linked inhibitor of apoptosis protein and receptor-interacting protein kinase 1, promotes cell apoptosis while reducing necrosis, and thereby effectively alleviates acute pancreatitis induced by caerulein combined with lipopolysaccharide 234. Transient transfection of siRNA to knock down GRP78 expression in p53-deficient PC-3 cells enhances methylseleninic acid (MSA)-induced activation of the UPR pathway, accompanied by UPR-driven proliferative arrest in tumor cells, thereby potentiating the anticancer activity of MSA in PC cells 235.

It can be seen that GRP78 exhibits remarkable versatility as a target for gene therapy, given that both overexpression and knockdown strategies can enhance therapeutic sensitivity and alleviate disease progression in corresponding disorders. Moving forward, efforts could be directed toward optimizing targeted delivery systems, combining GRP78-targeted gene therapy with chemotherapy or immunotherapy to improve therapeutic efficacy, and exploring its therapeutic potential in a broader spectrum of diseases (Figure 10D).

## Conclusions and perspectives

GRP78, a core molecular chaperone in the ERS pathway, plays a critical role in maintaining protein folding, cellular homeostasis and stress adaptation. Accumulating research has revealed that dysregulated expression and function of GRP78 are widely involved in the pathogenesis of multiple diseases, making it a target with great research significance and translational potential. An in-depth comprehension of its regulatory mechanisms is critical to the development of targeted therapeutic strategies. This review summarizes the structure, PTMs, and physiological functions of GRP78, as well as its roles in diverse disorders such as cancer, neurodegenerative diseases, infectious diseases, cardiovascular diseases, inflammatory diseases, and metabolic diseases. Furthermore, we summarize the latest compounds capable of regulating GRP78, including targeted and non-targeted modulators, to provide guidance for the future development of more effective GRP78-targeted modulators.

As a core ER molecular chaperone, the dynamic regulation of the structure and function of GRP78 permeates the entire process of cellular homeostasis maintenance and disease pathogenesis, playing a pivotal role in various disorders such as cancer and neurodegenerative diseases. However, GRP78 is not a simple “deleterious molecule” in diseases. For instance, in PD, its overexpression exerts a neuroprotective effect; in the early stage of MI/RI, the upregulation of GRP78 represents a critical endogenous cytoprotective response. In cancer, GRP78 not only promotes tumor survival and chemoresistance but also may participate in immune surveillance under specific circumstances. Its “double-edged sword” property and cross-disease regulatory capacity make it a highly promising therapeutic target, but also render the research and translation of GRP78 extremely complex. However, most current studies focus on developing broad-spectrum inhibitors, lacking disease-specific strategies that can precisely regulate GRP78 function. Given this duality, intervention strategies must possess extremely high disease-type specificity, stage specificity, and even cell-type specificity; thus, simple knockdown or overexpression may be counterproductive. For disorders such as AD and PD, enhancing the chaperone function of GRP78 could theoretically help eliminate misfolded proteins. However, direct and controllable small-molecule activators of GRP78 are currently lacking. BIX, the only known indirect activator, lacks specificity and has a complex mechanism of action. This research gap greatly limits the therapeutic neuroprotective potential of harnessing GRP78.

In recent years, artificial intelligence programs such as AlphaFold have been widely applied in the field of structural biology 236. Precisely predicting the conformational changes of GRP78 under different nucleotide states and ligand binding conditions via AlphaFold and other tools, combined with molecular dynamics simulations to identify potential allosteric activation pockets, can provide a structural basis for designing the first bona fide GRP78 small-molecule agonists. Furthermore, we know that the ATPase activity and functional cycle of GRP78 are precisely regulated by co-chaperone proteins. As mentioned earlier, Hsp40/DnaJ family proteins can activate its ATPase activity and accelerate the functional cycle; in contrast, such nucleotide exchange factors as GRP170/BAP promote ADP/ATP exchange to facilitate GRP78 resetting. Thus, molecules can be designed to promote the binding of GRP78 to activating co-chaperones or inhibit its interaction with negative regulators. We propose the development of “molecular glue”-type small molecules that do not directly bind to the catalytic center of GRP78 but specifically stabilize the PPI interface between GRP78 and specific positive co-factors. Enhancing such physiological activation complexes would comprehensively improve the folding capacity of the GRP78 network. However, it is first necessary to resolve the structures of key functional complexes such as GRP78-ERdj4, followed by fragment-based screening or virtual screening to identify compounds capable of binding to both proteins simultaneously and stabilizing their interface.

Targeted protein degradation has emerged as an exciting novel therapeutic strategy, particularly in the field of oncology. PROTAC technology has emerged as a research hotspot in recent years. By linking a target protein ligand to an E3 ubiquitin ligase ligand via a linker, this technology mediates the ubiquitination and proteasomal degradation of target proteins without relying on occupancy of the active site 237. Given the targeting capability and degradation-driven property of PROTACs, they could be considered for application in the functional regulation of GRP78. One end bind to GRP78 in specific inactive or modified states, while the other recruits an E3 ubiquitin ligase to selectively degrade these low-activity GRP78 molecules. ERS induced by reduced GRP78 levels promotes the initiation of the UPR, triggering the *de novo* synthesis of a large quantity of new GRP78 with intact activity to achieve functional “activation”. In 2025, the first Hsp70 interactome-mediated proteolysis targeting chimera (Hsp70-PROTAC) molecule, GDAz-3, was successfully developed. This molecule can efficiently degrade pathogenic proteins such as GPX4 via a dual degradation pathway and selectively trigger ferroptosis, making it useful for ferroptosis-driven cancer therapy 238. The success of this study is expected to provide valuable insights for the development of GRP78-targeting PROTAC molecules.

The previous text has systematically analyzed the transformation disorders of GRP78 inhibitors in terms of selectivity, long-term toxicity and patient stratification. On this basis, it is necessary to further emphasize that insufficient selectivity will directly magnify the long-term toxicity risk of normal tissues; the lack of stratified biomarkers related to the subcellular localization of GRP78 makes it difficult for even highly selective inhibitors to target effective populations in clinical trials. Therefore, it is necessary to determine the priority of the three major transformation obstacles. We believe that the most crucial issue at present is not the drug molecules themselves, but the absence of clinically detectable functional biomarkers. The absence of a stratified strategy will directly magnify the misjudgment of selectivity and toxicity issues. Imagine that if the patients recruited in a clinical trial do not fall within the effective range, even if the drug has perfect selectivity and controllable toxicity, no therapeutic effect can be observed. Perhaps the experience of PARP inhibitors that have been successfully clinically translated can be drawn upon to develop circulating biomarkers capable of reporting functional inhibition of GRP78. Relevant research has established a highly sensitive homogeneous polyADp-ribose (PAR) detection method based on the direct target product of PARP inhibitors, PAR, and accurately screened out all known PARP inhibitors in the compound library 239. As a member of the Hsp70 family, the core function of GRP78 is ATP-dependent substrate folding and release. The generation of ADP can be taken as the detection object, and an enzyme coupling method similar to PAR detection can be adopted to establish an *in vitro* detection system for the ATPase activity of GRP78. However, the ATPase activity of GRP78 is much lower than the catalytic efficiency of PARP, so a more sensitive detection platform is needed. In addition, some studies have developed a radiolabeled PARP inhibitor that can specifically bind to PARP proteins in tumors and can non-invasively monitor target status through PET imaging 240. PET imaging is used not only for patient screening but also for real-time monitoring of whether the drug actually reaches and occupies the target site. Given that GRP78 is expressed on the surface of various tumor cells and has specific conformational epitopes that can be recognized by antibodies or small molecules, the development of PET imaging probes targeting csGRP78 can achieve non-invasive, quantitative and dynamic target monitoring. At present, some novel probes targeting GRP78 have been developed, including ^18^F-labeled cyclic peptide probes 241, which are currently recognized as the most promising methods for clinical translation. In addition, there are ^68^GA-labeled linear peptide probes 242, antibody probes 243, and D-type polypeptide probes 244, all of which are relatively mainstream design schemes in recent years. In conclusion, the successful experience of PARP inhibitors indicates that functional biomarkers are not a single technology but a complete chain of evidence ranging from direct target products to downstream signaling networks. For the targeted therapy of GRP78, this logic can be drawn upon to establish the ability to “detect whether the target is inhibited”, which is not only a necessary condition for verifying the drug mechanism but also the scientific basis for determining the dosage and selecting patients in clinical transformation.

GRP78 is involved in maintaining ER homeostasis, and its basal functions are critical for cell survival. Thus, pan-inhibition of GRP78 activity not only eliminates pathological cells but also severely impairs the function of normal tissues, resulting in unavoidable off-target toxicity. This also constitutes a major hurdle to current clinical translation. Leveraging the characteristic of csGRP78 being specifically overexpressed in various cancer cells, we can consider developing antibody-drug conjugates or targeted delivery systems—linking highly active GRP78 modulators to csGRP78-targeting antibodies or aptamers to enhance the enrichment efficiency of modulators in pathological tissues. On the other hand, exosomes can be utilized as endogenous carriers for the targeted delivery of siRNA to silence GRP78 in pathological tissues, thereby minimizing systemic toxicity. Another interesting approach involves delving into the key modifying enzymes that drive GRP78 dysregulation in specific diseases. As previously mentioned, the phosphorylation, palmitoylation, and other modification sites of GRP78 under pathological conditions exhibit disease specificity. Thus, developing small-molecule inhibitors targeting these modification sites may reverse its pathogenic state without compromising the basal functions of GRP78.

Given that GRP78 serves as a convergence point of multiple stress pathways, its single-agent inhibition or activation may be counteracted by other compensatory pathways. Thus, combined targeting of GRP78 with key upstream and downstream nodes represents a more effective strategy. For instance, combining GRP78 inhibitors with agents targeting its compensatory pathways (e.g., autophagy inhibitors) can prevent cancer cell evasion. As previously noted, GRP78 regulates oncoproteins such as c-MYC, suggesting that its combination with epigenetic regulators (e.g., BET inhibitors) may elicit synergistic effects.

In summary, research on GRP78 is at a critical juncture transitioning from “mechanism discovery” to “precision intervention”. With the in-depth elucidation of pathological mechanisms of GRP78—particularly the clarification of its subcellular localization, PTMs, and disease-specific signaling pathways—it is expected to achieve a leap from pan-target inhibition to precision regulation, thereby providing novel therapeutic strategies for intractable diseases such as cancer and neurodegenerative diseases. Furthermore, the potential of GRP78 as a cross-disease regulatory target will also promote the advancement of universal technologies for broad-spectrum therapeutic interventions against multiple disorders, opening up new avenues for the precise treatment of human diseases.

## Figures and Tables

**Table 1:** Structural and functional differences between GRP78 and the representatives of the Hsp70 family.

Protein name	Main subcellular localization	The length of the flexible linker	Motifs contained in the C terminus	Substrate selectivity of SBD	Regulatory mechanism	Cofactors	Response to stress
GRP78	ER	10 aa	KDEL	Has high affinity for negatively charged peptide segments; opening and closing are regulated by the NBD nucleotide state	Expression induced by ERS	ER-resident DnaJ	Response to ERS
Hsp72	Cytoplasm	8 aa	EEVD	Prefers neutral hydrophobic peptide segments; opening and closing depend on auxiliary factors	Expression regulated by HSF	Hsp40, NEF, etc.	Response to heat shock, etc.
HSPA8	Cytoplasm, Nucleus	8 aa	EEVD	Prefers neutral hydrophobic peptide segments and specifically recognizes KFERQ-like motifs 245; opening and closing are regulated by the nucleotide state of NBD, and also depend on the cooperation of auxiliary factors	Expression is stable, ubiquitous	BAG, Hsp40, etc.	Response to heat shock 246, oxidative stress, etc.
HSPA9	Mitochondria	9 aa	EEKQ	Recognition of mitochondrial matrix proteins 247; opening and closing are regulated by the NBD nucleotide state and also depend on the cooperation of mitochondria-specific auxiliary factors	Tissue/tumor-specific transcriptional activation (such as ESRRA^248^)	DNLZ 249	Response to oxidative stress, etc. (not response to heat shock 250)
HSPA6	Cytoplasm, Nucleus	8 aa	EEVD	The opening and closing are allosterically regulated by the NBD nucleotide state, and the substrate selectivity is not yet clear	Induced expression by high temperature and other factors, no baseline expression (only present in the human genome)	BAG, Hsp40, etc.	Response to heat shock, etc.

**Table 2:** PTMs and regulatory roles of GRP78.

Modification type	Modification site	Enzyme	Effect on GRP78	References
Phosphorylation	Thr453, Thr473 and Thr500; Ser and Tyr residues (The specific residue(s) remain unidentified)	MEK	Leading to GRP78 inactivation, unable to bind substrates	24-30
Acetylation	Lys633	HDAC6	Causing GRP78 to be unable to sort into multivesicular bodies	53-55
ADP-ribosylation	Arg470, Arg492	hARTC1, cARTC2.1	Disrupting the binding of GRP78 to its substrate, reducing the stability of the complex	32-35
Methylation	Lys585	METTL21A	Lys585 trimethylation is a marker of steady-state GRP78, and promoter hypermethylation leads to reduced GRP78 mRNA and protein levels	48, 49
Ubiquitination	Lys48	UHRF1, GP78	Inhibiting GRP78 transcription and promoting GRP78 degradation	41, 42, 46
Deubiquitination	Lys48	OTUD3	Prolonging the half-life of GRP78 and enhancing its stability	43, 44
S-palmitoylation	Cys420	ZDHHC9	Enhancing GRP78 stability, maintaining its localization in the ER, and strengthening its inhibition of UPR sensors	47
S-sulfhydration	Cys420	CTH	Inducing the dissociation of IRE1α from GRP78	36-39
S-nitrosylation	Cys residue (The specific residue(s) remain unidentified)	NO group transfer-related enzyme	Inhibiting the ATPase activity and chaperone function of GRP78	50-52

**Table 3:** The roles of GRP78 in cancer.

Type of cancer		The effect of GRP78 on cancer	References
Solid tumors	PC	Activating PI3K/Akt and other pathways maintains cancer cell survival and metastasis; Its high expression is strongly associated with CRPC status.	83, 84
	BC	Activating the Wnt/β-catenin signaling pathway promotes cancer cell metastasis; Mediate estrogen deprivation resistance in combination with BIK.	89, 100
	Lung cancer	Activating of TGF-β/Smad2/3 signaling pathway and MEK1/2/ERK1/2 signaling axis promotes cell Metastasis; Activating of the PI3K/Akt pathway increases the invasive ability of cancer cells; Binding with OTUD3 enhances tumor-forming ability.	44, 86, 87
	CRC	Binding to STAT3 promotes cancer cell proliferation and metastasis; Enhance the invasive ability of CRC cells.	90, 97
	PDAC	Activating Akt signaling promotes cancer cell proliferation; Preserve the self-renewal and tumorigenic potential of pancreatic cancer stem cells; Activating the YAP/TAZ signaling axis enhances the migration and invasion capabilities of cancer cells; Mediate gemcitabine resistance.	80, 93, 99
	GBM	Mediate resistance to etoposide and cisplatin, and display resistance to γ-radiation.	101
	Bladder cancer	Palmitoylation of itself can promote drug resistance in cancer cells; Promote tumor cell survival, proliferation, and EMT, and enhance cell migration and invasion capabilities.	47, 88
	GC	Binding with Gal-1 promotes the proliferation, migration, and invasion abilities of GC cells; Mediate cancer cell drug resistance; Activating the VEGF/VEGFR2 pathway is involved in tumor angiogenesis.	91, 92
	OC	Binding with a specific fragment of LINC00662 promotes proliferation, invasion, and metastasis of OC cells.	98
	HNC	Closely related to the characteristics of cancer stem cells; Promote c-MYC protein expression and enhance cancer cell vitality.	94-96
Hematologic tumors	ALL	Overexpression can promote leukemia cell survival, migration, and infiltration, while inhibiting apoptosis; Maintain leukemia stem cell characteristics.	102
	AML	Its expression level is negatively correlated with sensitivity to FLT3 inhibitors; Activating the PI3K/Akt/mTOR signaling cascade promotes the proliferation of leukemia stem cells.	105, 106
	MM	Promoting autophagosome formation mediates cell resistance.	107

**Table 4:** GRP78-targeted small molecule and peptide modulators.

Compound	Structure	Target/Binding site	Effect on GRP78	Cells/animal models	Therapeutic applications	Ref.
VER-155008	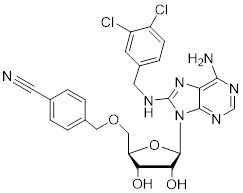	NBD IC50 = 2.6 ± 0.39 μM Kd = 0.2 μM	Inhibition	HT29 CRC cells, HCT116 cells, BT474 cells, MDA-MB-468 TNBC cells, 5×HCT116 tumor xenograft immunodeficient mice	CRC, BC	178, 179
HA15	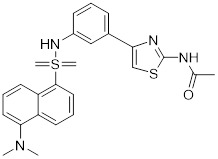	NBD	Inhibition	Cell models ≥20 types, including A375 cells, patient-derived primary melanoma cells, etc.; 36×melanoma xenograft immunodeficient mice	Melanoma	181
PST	PEGKGEQEHSQQKEEEEEMAVVPQGLFRG-NH2 (seq)	NBD IC50 ≈ 5.2 μM	Inhibition	Human HepG2 liver cells, mouse 3T3-L1 adipocytes; 3×CHGA⁻/⁻ mice	T2DM, obesity-related metabolic disorders	182
FL5	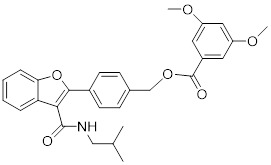	NBD ∆Tm rose by 2.65 ℃	Inhibition	HUVECs, human renal carcinoma cells 786-O, mouse fibroblasts Swiss-3T3	Kidney cancer	183
EGCG	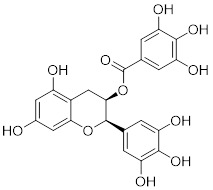	NBD Kd = 0.7 μM	Inhibition	Malignant glioma cell lines, CRC cell lines, etc.; 24×DLD1 CRC xenograft model mice	CRC, GBM	185-187
Sanguinarine	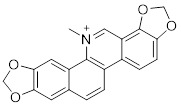	NBD IC50 = 8.6 μM	Inhibition	MCF-7 human BC cells	BC	188
GRP78-IN-3	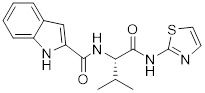	SBD IC50 = 0.59 ± 0.06 μM	Inhibition	There are 9 types of cell models, including HCT116 cells, A549 non-small cell lung cancer (NSCLC) cells, MDA-MB-231T cells, etc.	Lung cancer, CRC, BC, etc.	189
YUM70	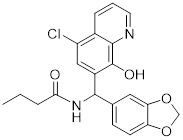	SBD IC50 = 1.5 ± 0.3 μM	Inhibition	26 cell models, including MDA-MB-231 cells, PANC-1 cells, etc.; 10×MIA PaCa-2 pancreatic cancer xenografts in immunodeficient mice	BC, pancreatic cancer, etc.	14, 95
HM03	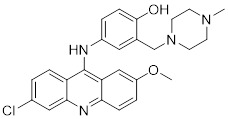	SBD	Inhibition	HCT116 cells	CRC	190
Bag-1 peptide	/	SBD IC50 = 2.6 ± 0.5 μM Kd = 5.7 ± 0.8 μM	Inhibition	PC cell line; 15×PC xenograft model mice	PC	191
Inauhzin-C	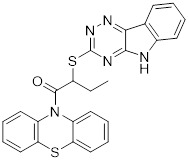	NLS Kd = 12.73 ± 0.468 μM	Inhibition	H460 cells, SK-MEL-147 metastatic melanoma cells, PLC5 HCC cells, etc.	NSCLC, melanoma, HCC, etc.	192
Parishin	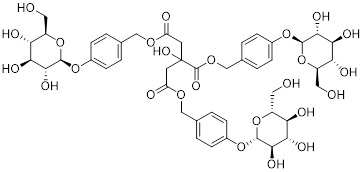	NLS Kd = 3.52 μM	Inhibition	HK-2, NRK-52E renal tubular epithelial cells, HEK293T cells; 24×db/db mice	DN	42

**Table 5:** Non-targeted small molecule modulators of GRP78.

Compound	Structure	Target/mechanism of action	Effect on GRP78	Cells/animal models	Therapeutic applications	Ref.
Bortezomib	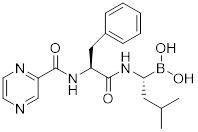	20S proteasome, tissue protease L/B	Activation	MCF-7 cells, T-47D cells, TR5 cells	ER-positive BC	197
Carfilzomib	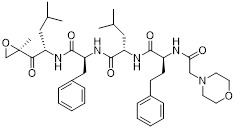	20S proteasome	Activation	NCI-H520 NSCLC cells, A549 cells, etc.	NSCLC	198
Atazanavir	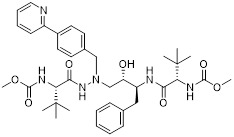	20S proteasome	Activation	10×GBM model mice	GBM	199
Vorinostat	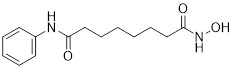	Lys585	Acetylation	GBM cell lines such as U251, and PC cell lines such as DU145	GBM, PC	200
Panobinostat	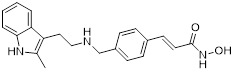	HDAC6	Acetylation	MCF-7 cells, MDA-MB-231 cells	BC	54
Sorafenib	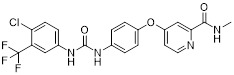	ERS→ RE1α/JNK→GRP78/CD44 membrane translocation→CD44-GRP78-IGF1R complex→PI3K/Akt pathway activation	Inhibition	HCC cell lines such as SNU449; 15×HCC xenograft model mice	HCC	201, 202
Regorafenib	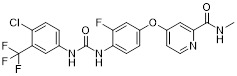	GRP78 itself (induces its conformational change/promotes ubiquitination modification)	Inhibition	GBM5 and other GBM cell lines; 15×immunodeficient nude mice	GBM	202
Abemaciclib	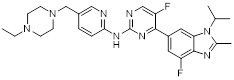	NBD (Predicted binding energy -10.45 kcal/mol)	Inhibition	/	/	204
Ribociclib	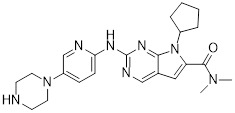	NBD (Predicted binding energy -8.75 kcal/mol)	Inhibition	/	/	204
Crizotinib	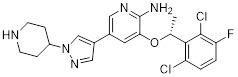	SRC activation→GRP78 membrane translocation→Par-4-csGRP78	Membrane translocation	Human lung cancer cell lines, mouse lung cancer cell lines; 20×human lung cancer cell xenograft model mice, 20×syngeneic mouse lung cancer cell (KP7B) transplant model mice	ALK-negative NSCLC	203
Curcumin	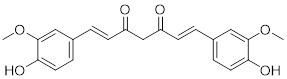	Arg297, Ser300, Arg367 (binding energy -8.5 kcal/mol)	Inhibition	18×Irinotecan-induced intestinal mucosal injury in mice	Intestinal mucosal injury	205-207
Quercetin	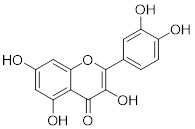	GRP78 itself (specific site not yet clear)	Inhibition	Nthy-ori-3-1 non-tumorigenic thyroid cells; 40×hyperuricemia-induced chronic kidney disease model mice	Thyroid injury, CKD	208, 209
Resveratrol	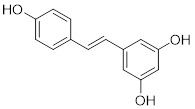	ERS-iROS Axis	Activation	Neuro-2a, NB41A3 mouse neuroblastoma cells; 24×neuroblastoma xenograft mice	Neuroblastoma	210
Berberine	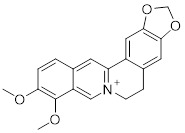	ATF6, AMPK, FAK	Inhibition (CRC, intracranial aneurysm)/Activation (liver cancer)	Human CRC cell lines, RAW264.7 mouse macrophages, HepG2 cells; 36×intracranial aneurysm model mice	CRC, intracranial aneurysm, liver cancer	211-213
Sanguinarine	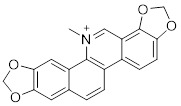	ROS→ERS→UPR activation→GRP78 upregulation	Activation	SPC-A1 human lung adenocarcinoma cells	lung adenocarcinoma	214
Shikonin	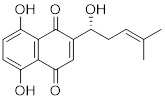	Mitochondrial Ca²⁺ accumulation→ERS→UPR activation; TLR4/NF-κB pathway inhibition→reduction of inflammation/fibrosis→ERS→UPR activation	Activation	SNU-C5/5-FUR human 5-FU-resistant CRC cells, H9C2 human cardiomyocytes; 60×mice with myocardial injury induced by ISO	5-FU resistant CRC, ISO-induced myocardial injury	215-217
Celastrol	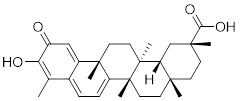	Cys41 (binding energy -8.1 kcal/mol) / GLUT1	Inhibition	RAW264.7 mouse macrophages, 4T1 Fluc mouse TNBC cells, MDA-MB-231 human TNBC cells; 80× diet-induced obesity model mice, 40× orthotopic BC model mice	Diet-induced obesity, TNBC	218, 219
Ginsenoside Rg1	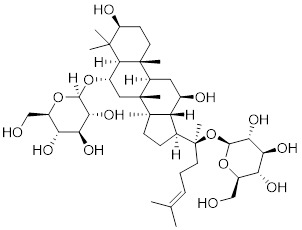	GRP78 itself (specific site not yet clear)	Inhibition	80×diabetic cardiomyopathy model mice	DCM	220, 221
Ginsenoside NGR1	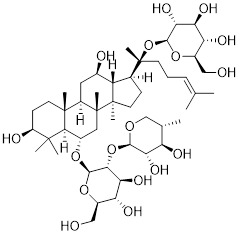	GRP78 itself (specific site not yet clear)	Inhibition	H9c2 rat cardiomyocytes; 100×mice with isolated heart I/R model at magnification	MI/RI	221, 222
Ginsenoside Rg3	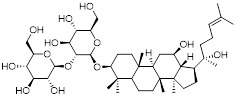	GLUT1	Inhibition	4T1 Fluc mouse TNBC cells, MDA-MB-231 human TNBC cells; 40× orthotopic BC model mice	TNBC	219

## Abbreviations

5-FU: 5-fluorouracil
α_2_M*: Activated α_2_-macroglobulin
α-syn: α-synuclein
ACC: Acetyl-CoA carboxylase
ACPA: Anti-citrullinated protein antibodies
AD: Alzheimer’s disease
ALL: Acute lymphoblastic leukemia
ALS: Amyotrophic lateral sclerosis
AML: Acute myeloid leukemia
ATF6: Activating transcription factor 6
BC: Breast cancer
CAFs: Cancer-associated fibroblasts
COPD: Chronic obstructive pulmonary disease
CPT1A: Carnitine palmitoyltransferase 1A
CRC: Colorectal cancer
csGRP78: Cell surface GRP78
CT: Cholera toxin
CTA: Cholera toxin’s A subunit
CTL: Cytotoxic T lymphocyte
DA: Copaminergic
DC: Dendritic cell
DCD: Dermcidin
DCM: Diabetic cardiomyopathy
DENV: Dengue virus
DM: Diabetes mellitus
DN: Diabetic nephropathy
DSS: Dextran sulfate sodium salt
DTT: Dithiothreitol
EF: Edema factor
EGCG: Epigallocatechin gallate
EMT: Epithelial-mesenchymal transition
ER: Endoplasmic reticulum
ERAD: Endoplasmic reticulum-associated degradation
ERS: Endoplasmic reticulum stress
FAK: Focal adhesion kinase
FASN: Fatty acid synthase
FDA: the U.S. food and drug administration
Gal-1: Galectin-1
GBM: Glioblastoma
GC: Gastric cancer
GLS1: Glutaminase 1
GLUT4: Glucose transporter 4
GPI: Glycosylphosphatidylinositol
GRP78: 78 kDa glucose-regulated protein
HBV: Hepatitis B virus
HBX: the X protein of HBV
HCC: Hepatocellular carcinoma
HCMV: Human cytomegalovirus
HCV: Hepatitis C virus
HD: Huntington’s disease
HDAC6: Histone deacetylase 6
HNC: Head and neck cancer
HSF: Heat shock factors
Hsp70: Heat shock 70 kDa protein
Hsp72: Heat shock-related 70 kDa protein 2
HSPA8: Heat shock protein family A member 8
HUVECs: Human umbilical vein endothelial cells
IA: Intracranial aneurysms
IBD: Inflammatory bowel disease
INZ-C: Inauhzin-C
IPC: Ischemic preconditioning
IRE1α: Inositol-requiring enzyme 1 alpha
ISO: Isoproterenol
KSHV: Kaposi’s sarcoma-associated herpesvirus
LDL: Low-density lipoprotein
LF: Lethal factor
MEK: Mitogen-activated protein kinase
mHtt: Mutant huntingtin
MI/RI: Myocardial ischemia/reperfusion injury
MM: Multiple myeloma
MSA: Methylseleninic acid
NAFLD: Non-alcoholic fatty liver disease
NBD: Nucleotide-binding domain
NGR1: Notoginsenoside R1
NLS: Nuclear localization signal
NPSLE: Neuropsychiatric systemic lupus erythematosus
NSCLC: Non-small cell lung cancer
PA63: Protective antigen 63 kDa fragment
PAR: PolyADp-ribose
PD: Parkinson’s disease
PDAC: Pancreatic ductal adenocarcinoma
PDX: Patient-derived xenograft
PERK: Protein kinase R (PKR)-like endoplasmic reticulum kinase
PiD: Pick’s disease
PP2A: Protein phosphatase 2A
PPI: Protein-protein interactions
PrPC: Normal cellular prion proteins
PrPSc: Scrapie Prion Protein
PSA: Prostate-specific antigen
PTMs: Post-translational modifications
RA: Rheumatoid arthritis
rAAV: Recombinant adeno-associated virus
ReA: Reactive arthritis
rfhsp-D: Recombinant fragment of human surfactant protein D
ROS: Reactive oxygen species
SBD: Substrate-binding domain
SCD1: Stearoyl-CoA desaturase 1
SFO: Subfornical organ
SMCs: Smooth muscle cells
SNc: Substantia nigra pars compacta
SOD1: Superoxide dismutase 1
SREBP1: Sterol regulatory element-binding protein 1
T2DM: Type 2 diabetes mellitus
TDP-43: TAR DNA-binding protein 43
TH: Tyrosine hydroxylase
TLR-3: Toll-like receptor 3
TME: Tumor microenvironment
TNBC: Triple negative breast cancer
Treg: Regulatory T cell
UC: Ulcerative colitis
UPR: Unfolded protein response
VEGF-B: Vascular endothelial growth factor B
